# Who is exposed and who is harmed? Social disparities in flood exposure and impact in Pernambuco, Brazil

**DOI:** 10.1007/s11111-026-00523-z

**Published:** 2026-04-21

**Authors:** Mohamad AlAbbas, Emily Hannum, Jere Behrman, Leticia Marteleto

**Affiliations:** Population Studies Center, University of Pennsylvania, Philadelphia, United States

**Keywords:** Environmental stratification, Flash floods, Extremely high precipitation, Natural disasters, Brazil

## Abstract

How do geospatial sorting and social stratification condition natural-disaster exposure and impact? Using the case of the May 2022 flash floods in Pernambuco, Brazil, we address this question with a dual-source approach that captures disparities in both exposure to anomalously high rainfall and the reported impacts of resulting floods. While exposure to anomalous rainfall is tied to geospatial segregation patterns, reported impacts may also reflect localized infrastructure, such as drainage systems or piped water availability, that capture municipality-level investment and capacity to withstand flooding. Findings from exposure analysis reveal significant disparities by race, religion, maternal education, and income. However, these relationships fully attenuate after adjustment for municipal location, suggesting that settlement patterns and geospatial marginalization drive the observed exposure disparities. Findings from the analysis of self-reported flood impact also show disparate impacts by race, employment status, number of children under 15, and household income, but in this case, only racial disparities are fully attenuated after spatial adjustments. Further findings show that low-income households and those with more children report significantly greater impacts even after adjusting for both municipal fixed effects and hazard exposure. Interaction models reveal steeper dose-response relationships between rainfall and impacts for low-income households, and we find evidence that household infrastructure significantly mediates economic disparities in flood impacts. Combined, results demonstrate that relying solely on externally measured exposure to gauge the stratified impacts of extreme precipitation or flash floods may miss important socioeconomic disparities. Both geospatial segregation patterns and household-level capacities to withstand environmental shocks shape vulnerability.

**Supplementary Information:**

The online version contains supplementary material available at 10.1007/s11111-026-00523-z.

## Introduction

Climate change is intensifying the frequency and severity of extreme-weather events, with profound consequences for human well-being, economic stability, and social inequality (Pecl et al., 2017; Wasko et al., 2021). Among natural hazards, floods are the most widespread and destructive, responsible for more disaster-related deaths and damages than any other hazard type (Ritchie et al., 2024; Statista, 2023; WMO, 2015). Moreover, floods can expose and amplify existing inequalities, disproportionately burdening the poor, the marginalized, and those living in precarious environments (Smith et al., 2022). In this study, we investigate how socioeconomic inequalities shape patterns of exposure to extremely high precipitation and patterns of impact in the aftermath of flooding. Using the 2022 flash floods in Pernambuco, Brazil as a case study, we adopt a dual-source approach that combines satellite-based measures of anomalous rainfall with individual survey reports to assess how social stratification may affect both exposure to heavy rainfall and, perhaps differentially, those who report the greatest losses.

Floods arise from a complex interplay of atmospheric and hydrological conditions. Historically, they were often gradual and predictable, characterized by slowly rising water levels during winters and driven by large-scale atmospheric fluxes (Meyer et al., 2022). Since the 1990 s, however, their frequency has doubled in Europe, with comparable increases observed globally (Owen et al., 2018). A major driver of this shift is global climate change, which has reshaped the hydrological cycle and intensified the frequency and severity of extreme rainfall events that trigger flash floods (Marchi et al., 2010; Markowski & Richardson, 2011). Such events are typically convective in origin, produced by slow-moving storm systems that release intense rainfall over sustained periods (Markowski & Richardson, 2011). When compounded by storm recurrence, back-building^1^, and saturated soils, these processes can amplify rainfall totals and trigger a destructive kind of flash floods called pluvial floods^2^ (Marchi et al., 2010; Meyer et al., 2022). Pernambuco’s May 2022 flood illustrates these dynamics. In just six days, the metropolitan region of Recife (MRR) received 140 mm more rainfall than the historical average for the entire month of May (Marengo et al., 2023). Linked to eastern wave disturbances, significant cold fronts, and slow-moving storms, this anomalous rainfall overwhelmed natural and built drainage systems, producing catastrophic flooding (Marengo et al., 2023).

While atmospheric, hydrological, and infrastructural conditions explain when and where floods occur, they do not by themselves capture the social dimensions of who is most affected. Disaster risk reduction (DRR) research offers a useful framework for this task, distinguishing among five dimensions: hazard, a potentially damaging physical event such as anomalous high rainfall; exposure, the presence of people or assets in harm’s way; vulnerability, the propensity of those exposed to suffer harm; risk, the latent potential arising from the intersection of exposure and vulnerability; and impact, the realized losses and disruptions (Cardona, 2004; Cardona et al., 2012). This framework underscores that disasters are not the product of hazard alone, but of hazard meeting vulnerability and exposure. Yet these dimensions largely describe the overall risk, not the social, cultural, and political-institutional processes that concentrate exposure and vulnerability (Imperiale & Vanclay, 2024). In line with social-resilience thinking and the Sendai priority to ‘understand risk in all its dimensions’ (UNDRR, 2015), we adopt a sociological lens that specifies how socioeconomic characteristics pattern who is exposed, who is vulnerable, and who bears the brunt of impact (Imperiale & Vanclay, 2021; Marchezini, 2020; Smith et al., 2022).

A sociological perspective emphasizes how social-structural inequalities shape each dimension of the DRR framework. Link & Phelan’s (1995) theory of fundamental causes reframes vulnerability as rooted in the unequal distribution of resources, such as income, education, or institutional access, that determine households’ capacities to anticipate, absorb, and recover from shocks. From this vantage point, disadvantaged groups may experience greater harm not simply because they are more exposed, but also because they lack the buffers needed to mitigate and recover from losses.

The concept of stratified sensitivity as framed by Torche and Nobles (2024) further suggests that even when hazard and location are held constant, more disadvantaged households may still experience greater impacts due to cumulative disadvantages, producing unequal outcomes under identical current conditions. As Raker (2025) emphasizes, exposure itself is socially produced: infrastructure, land markets, and policy legacies distribute risk unevenly, relegating some groups to precarious terrain while protecting others. Taken together, these insights move beyond the DRR framework’s dimensions to show that hazard, exposure, vulnerability, risk, and impact are also socially stratified.

These insights guide our empirical analysis of the May 2022 floods in Pernambuco, Brazil as a case study of how social-structural inequalities are reflected in the consequences of a flash flood. Pernambuco is emblematic of broader patterns in the Global South in which rapid urbanization, informal settlements, and spatial segregation interact to heighten disaster vulnerability (Assis Dias et al., 2020; Pereira Santos et al., 2022; Rasch, 2017). To disentangle the dynamics of flood exposure and impact in this setting, we adopt a dual-source approach. We use satellite-derived precipitation anomalies from the Climate Hazards Group InfraRed Precipitation with Station Data (CHIRPS) dataset (Funk et al., 2015) as measures of exposure. With CHIRPS, we map where anomalous rainfall occurred and individual-level exposure to excessive rainfall that resulted in flash floods. We then compare stratification patterns in anomalous-rainfall exposures with individual-level reports of impacts using panel survey data from the Demographic Consequences of Zika and Covid-19 Crises (DZC) project.^3^

Through our dual-source approach, we isolate two key mechanisms of environmental inequality: stratification of exposure, where some groups are disproportionately located in hazard-prone areas; and stratification of impacts, where disadvantaged groups report greater losses due to differences in resources, household composition, or infrastructure, even after controlling for exposure. Modeling interaction effects between precipitation and household characteristics further allows us to assess stratified sensitivity, whether disadvantaged groups report steeper marginal impacts as rainfall intensifies.

Ultimately, this work addresses how socioeconomic inequalities at both the individual and community levels shape exposure to extreme precipitation and the reported severity of its impacts in the aftermath of a flash flood. Specifically, we examine whether patterns of social stratification appear similarly in earth observation-based measures of rainfall exposure and in self-reported flood impacts, and to what extent disparities in both can be explained by residential location and socioeconomic residential segregation. We also ask whether social inequalities in self-reported impacts persist even after adjusting for geography and rainfall exposure, which would indicate the presence of additional, non-spatial vulnerabilities that influence how floods are experienced. Finally, we assess whether disadvantaged groups show greater sensitivity to very high precipitation, with equivalent increases in exposure producing disproportionately higher impacts among poorer, less-educated, or otherwise-more-vulnerable households. This final analysis also examines whether variation in household infrastructure, proxied by piped-water connectivity, helps explain differences in reported flood impacts among otherwise similar households.

The remainder of the paper is organized as follows. We first introduce the theoretical and contextual background of the study. We then describe our data and methods, outlining the Pernambuco field site, the survey and environmental datasets, and our analytical strategy. Next, we present results on stratification of exposure, impact, and sensitivity, before concluding with a discussion of the broader implications for research on social inequality and environmental disasters.

## Conceptual framework

We engage with the DRR framework, emphasizing that each of the five dimensions – hazard, exposure, vulnerability, risk, and impact – is shaped by long-standing social structures that allow for the permeation of inequality. In Recife, as in many urban centers of the Global South, floods are not merely hydrometeorological incidents but socio-spatial processes: residential sorting, infrastructural provision, and governance determine who resides in flood-prone areas, who benefits from protective systems, and who ultimately bears the costs when those systems fail.

From a sociological standpoint, vulnerability is relational rather than purely physical fragility. Following Link and Phelan (1995), unequal access to “flexible resources”, such as income, education, information, and institutional support, condition households’ capacities to anticipate, absorb, and recover from shocks. Two households facing an identical hazard may diverge sharply in impacts depending on whether they can mobilize savings, secure temporary shelter, or rely on institutional support (Elsässer & Schäfer, 2023; Erikson, 2015). Inequality operates across scales. At the macro-spatial level, segregation, land markets, and policy histories push marginalized groups into floodplains, steep slopes, or drainage‐deficient peripheries, producing stratified exposure (Gourevitch et al., 2022; Hendricks & Van Zandt, 2021; Rasch, 2017). At the micro‐social level, stratified sensitivity means that, holding hazard and location constant, disadvantaged households experience steeper impact gradients as intensity rises due to cumulative disadvantages, caregiving burdens, health constraints, and fewer buffers. (Brouwer et al., 2007; Khalid et al., 2025; Torche et al., 2024; Torche & Nobles, 2024)

Crucially, risk in DRR research is not just proximity to hazard; it is the social production of space. Political economy, and related patterns of infrastructure development and governance, distribute protections unevenly, shaping who occupies safer ground and who is relegated to precarious terrain with limited state presence. As Raker (2025) emphasizes, space itself reflects and reinforces inequalities. This observation motivates our empirical strategy of separating “where people live” (municipal fixed effects) from “who people are” (individual socioeconomic characteristics), allowing us to assess whether disparities are place-driven, people‐driven, or both. Infrastructure further mediates impact: systems such as water and electricity are often the first to fail under stress, and the reliability of infrastructure is strongly correlated with social position and residential formality (Mazele & Amoah, 2021; Nduhuura et al., 2021; Sänger et al., 2021; VanDerslice, 2011). In Pernambuco, households with piped water reflect greater infrastructural integration and state presence, while those without face compounded risks (Coleman et al., 2020).

Turning to the hydrological dimension, multiple definitions of flash floods have been used within the literature. In this study, we examine pluvial floods, which are sudden, independent of nearby bodies of water, and are triggered when short periods of intense rainfall overwhelm the absorptive capacity of soils and built drainage systems (Meyer et al., 2022). However, intense rainfall is rarely sufficient or singularly responsible for a flash flood. Rather, high antecedent soil moisture from preceding rains often saturates the soil, making it incapable of absorbing the sudden intense rainfall and setting the stage for rapid runoff once the drainage systems are overwhelmed (Marchi et al., 2010). Fully examining pluvial floods requires accounting for antecedent soil moisture and deviations from local rainfall climatology, not merely the moment of onset. In just six days, the capital city of Recife received 551 mm of rainfall, or 140 mm above the historical monthly average, with the heaviest downpours occurring on May 28 and linked to easterly wave disturbances and a cold front moving inland (Marengo et al., 2023). These conditions produced the city’s most devastating flood since 1966, killing more than 130 people and displacing tens of thousands (Davies, 2022; Marengo et al., 2023). Yet the hydrometeorological drivers alone do not explain why losses were so severe. As Marengo et al. (2023) document, Recife’s topography, which is marked by steep slopes and low-lying floodplains, combined with decades of unplanned expansion and insufficient-drainage infrastructure to channel extreme precipitation into catastrophic flooding.

While Recife bore the brunt, flooding extended across the state, where entrenched vulnerability amplified impacts for low-income households. The state has experienced rapid but uneven urban development that has produced entrenched-vulnerability traps: low-income households are priced out of safe, serviced areas and into risk‐prone sites, then face constrained response capacity when floods arrive (Rasch, 2017; Ribeiro et al., 2018; Wink Junior et al., 2024). Evidence links inequality to spatial segregation, under‐provision of public works, and differential adaptive capacity. In this way, there is an institutional pathway from income inequality to elevated vulnerability that aligns closely with DRR’s emphasis on capacities and susceptibilities (Al-Maruf et al., 2021; Carvalho & Netto, 2023).

## Data & Methods

### Field site

In Brazil, unequal patterns of development and regional disparities lead to differential levels of vulnerability to the impacts of flooding, especially in poorer areas like Pernambuco (Assis Dias et al., 2020; Pereira Santos et al., 2022; Rasch, 2017). This study site is situated in the coastal State of Pernambuco, in the northwestern region of the country. Pernambuco is home to the fifth-largest population center in Brazil, known as the MRR, which includes the state capital and 13 other municipalities (Baltar de Souza Leão et al., 2021).

**Fig. 1:**
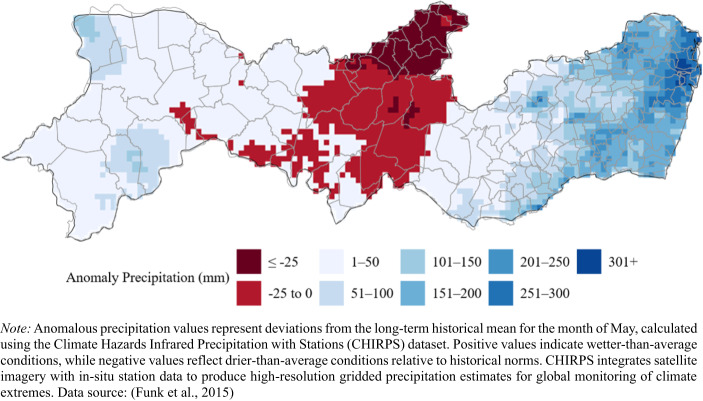
Anomaly Precipitation as estimated by CHIRPS across the State of Pernambuco

According to meteorological agencies of Brazil and Pernambuco, the MRR received more than 70% of the typical May rainfall in less than 24 h between May 27 and May 28 (Marengo et al., 2023). The heavy rain triggered a series of landslides and floods across the state, resulting in 130 fatalities, nearly 4,200 homeless people, and some 600 people displaced in the MRR (Davies, 2022; DW, 2022; Marengo et al., 2023). These extreme precipitation levels are visually represented in Fig. 1, which shows the anomaly precipitation estimates from CHIRPS across all regions of Pernambuco in May 2022.

Figure 1 illustrates the spatial distribution of precipitation anomalies, highlighting extreme deviations from normal rainfall levels. The darkest blue regions, which received up to 339 mm more rainfall than the historical May average for those areas, are concentrated in the eastern part of the state, particularly around Recife and its surrounding municipalities, coinciding with the region’s most severely impacted by flooding and landslides. The map also reveals that while some western areas experienced moderate anomalies, the coastal region bore the brunt of the excessive rainfall. This spatial pattern aligns with reports of widespread urban damage and displacement concentrated in the MRR (DW, 2022; Marengo et al., 2023).

### Data

Our dual-source approach distinguishes between two interrelated processes: Stratification of exposure, whereby some groups are disproportionately located in high-risk zones, and stratification of impacts, whereby disadvantaged households report greater disruptions even when exposed to similar-hazard conditions. This approach requires the combination of several sources of data.

#### Hazard and exposure data

To quantify physical exposure to the May 2022 flash floods, we use data from the CHIRPS dataset (Funk et al., 2015), which provides high-resolution (approximately 5 km) gridded-precipitation estimates by blending geostationary-infrared observations with ground-gauge data against a long-run climatology. We note that CHIRPS does not explicitly factor sub-kilometer topography, urban drainage networks, or micro-convective bursts. We operationalize^4^ exposure through precipitation anomalies for May 2022, defined as the deviation of May 2022 precipitation from the long-term average of all Mays since 1981. Anomalies capture departures from expected seasonal patterns and offer a standardized measure of rainfall extremity. Though the floods occurred at the end of May, we use the precipitation for the whole month because conditions before the floods, in particular soil saturation, are important preconditions for floods. Focusing on anomalies—rather than absolute rainfall—addresses several considerations. Absolute totals are less informative in regions like Pernambuco, where seasonal rainfall is normally high and communities are accustomed to managing routine precipitation; what overwhelms hydrological and social systems are departures from these ecological baselines (Marengo et al., 2023). Anomalies also align conceptually with the idea of environmental shocks as disruptions to ecological and social norms, which is consistent with sociological framings of disaster as the breakdown of expected routines. Finally, anomalies better reflect social perceptions: households typically interpret extreme rainfall not in absolute millimeters but in terms of how unusual, unexpected, and disruptive it feels compared to the “normal” seasonal cycle. For these reasons, and following evidence from Marengo et al. (2023), we treat rainfall anomalies as the most appropriate measure of hazard exposure for comparing with household reports of flood disruption.

Anomalous rainfall alone, however, does not fully determine flood hazards. Whether extreme rainfall translates into damaging floods depends critically on antecedent and environmental conditions (Meyer et al., 2022). Soil moisture plays a key role: high pre-event saturation limits infiltration capacity, accelerating surface runoff and amplifying flood severity even when rainfall totals are comparable for two periods. Land-use patterns also shape runoff dynamics; yet in our sample, over 95% of respondents reside in urban environments, leaving insufficient variation to include land cover as a meaningful covariate. Instead, we incorporate elevation and rainfall jointly—both as components of our exposure-adjusted impact analysis and as diagnostic tools for examining spatial segregation. Elevation helps distinguish which populations inhabit flood-prone lowlands or landslide-prone slopes, allowing us to assess whether spatial inequalities align with socioeconomic status.

#### DZC survey data and analytical sample

To assess the social experience of flood disruption, we use the DZC Project. Initiated in 2020, the DZC is a panel of women ages 18–34 in Pernambuco (eight waves collected in 2020–2022 and four ongoing), a state marked by repeated public-health and environmental crises. The goal of the project is to understand reproductive and demographic responses to Zika and COVID-19. Because of the consequential impacts of the May 2022 floods, the project added a module on their impact.

Wave 3, conducted after the flood, included four flood-related questions on material loss, housing disruptions, service interruptions, and neighborhood flood inundation. These questions allow us to capture flood experience at the household level, providing a socially situated measure of flood impact. By linking these responses to respondent characteristics collected in earlier waves, we can analyze how pre-existing social conditions shape perceived flood impacts. The analytical sample includes 1,411 women who participated in all three waves^5^ and provided valid responses to the flood questions and core-stratifying variables: race, education, maternal education, household income and size, employment status, religion, and number of children. All respondents were residing in Pernambuco at the time of the flood, providing a unique population-based lens on how environmental shocks interact with social inequalities in a high-risk setting.

### Measures

This study draws on a series of household- and individual-level measures that align with the theoretical frameworks of fundamental-social causes, stratified sensitivity, and socio-spatial vulnerability. Each measure is not only conceptually grounded but also empirically salient in the Brazilian context, where entrenched inequalities, fragmented urban development, and uneven public services amplify stratified disaster risks. Embedding these contextual dynamics into our operationalization of variables allows us to test whether reported flood outcomes in Pernambuco reflect broader social hierarchies (Table 1).

**Table 1:** Descriptive statistics of analytical sample, characteristic, and environmental variables

	Summary
*N*	1,411
Anomaly Precipitation (mm)	243.990 (49.561)
Elevation	59.917 (125.865)
Soil Moisture	452.152 (79.972)
Z-score vs. 1981–2021 6-day means (May 25–30)	5.332 (1.524)
Reported Impact Score	0.000 (1.522)
Age (2022)	29.379 (4.909)
Current Number of Children < 15	0.760 (0.930)
Race	
White	408 (28.9%)
Non-White	1,003 (71.1%)
Religion	
Catholic	509 (36.1%)
Non-Pentecostal Evangelical	260 (18.4%)
Pentecostal Evangelical	269 (19.1%)
Other religion	105 (7.4%)
Atheist/Agnostic	268 (19.0%)
Respondent’s Mother’s Education	
Less than Highschool	626 (44.4%)
Highschool	514 (36.4%)
Some College or More	271 (19.2%)
Respondent’s Education	
High-school or Less	816 (57.8%)
Some College or More	595 (42.2%)
Employment Status	
Formal Employment	710 (50.3%)
Informal Employment	263 (18.6%)
Out of the Labor Force	438 (31.0%)
Household Income	
1/2 Minimum Wage or Below	135 (9.6%)
1/2 to 1 Minimum Wage	296 (21.0%)
1 to 2 Minimum Wage	355 (25.2%)
2 to 3 Minimum Wage	252 (17.9%)
3 Minimum Wage or Above	373 (26.4%)
Frequency of Water Supply Outage	
Never	1,094 (78.0%)
At Least Once a Week	309 (22.0%)

Based on data from the restricted-access version of the DZC survey

*Precipitation Anomaly* captures the millimeter (mm) positive deviation in rainfall for May 2022 relative to the long-term historical average of all Mays since 1981, as estimated by the CHIRPS dataset. This continuous measure reflects localized rainfall extremes at the 4.7 km^2^ grid-cell level, all of which were positive, reporting excess rainfall, and is mapped to respondent GPS coordinates to estimate household-level exposures. Anomalies are used rather than absolute precipitation totals to better approximate the shock value of extreme weather events. The sample mean anomaly is 244 mm, with values ranging from 24 mm to 332 mm. 

*Soil Moisture* indexes antecedent saturation that governs how additional rainfall is partitioned into infiltration versus rapid runoff and localized flooding. We use monthly soil moisture for May (the storm’s month) from NOAA’s CPC product at 0.5° resolution, mapped to respondent GPS locations. Values are reported in the dataset’s native volumetric units ($\:{\mathrm{m}}^{3}$); in our analytic sample, soil moisture averages 452 (SD = 80) on the CPC scale.

*Elevation* indicates meters above sea level derived from AWS Terrain Tiles (Larrick et al., 2020) at ~ 2 m resolution and extracted at respondent coordinates. Elevation provides a first-order topographic control on flow accumulation and drainage efficiency. In our data, the mean elevation is 60 m (SD = 125 m).

*Reported Impact Score* is constructed from four ordinal DZC survey questions capturing multiple dimensions of household disruptions: (1) “Lack of water, electricity, internet, or cellphone signal for at least one day,” (2) “Loss of goods such as automobiles, motorcycles, cell phones, or appliances,” (3) “Loss of housing or becoming homeless,” and (4) “Flooding at home, in the building, on the street, or in the neighborhood.” Each was coded on a 0–5 scale, where 0 indicates no effect, and 5 indicates a severe effect. To derive weights for our index, we applied principal component analysis (PCA). The first principal component explained 58% of the variance and loaded positively on all items (0.45–0.54): loss of goods (0.54) and homelessness (0.52) loaded most strongly, followed by loss of connection (0.47) and flooding (0.45). We use this component as our *Reported Impact Score*. This measure reflects the shared variance across disruptions while weighing most heavily the forms of loss that covary. 6

*Age* is included as a continuous control to account for variation in reported impact by life stage, with an average respondent age of 29 years in 2022. *Number of Children Under 15* captures household composition, a known marker of vulnerability. Families with more children often face greater logistical burdens and higher emotional and economic strain during crises. In Brazil, recent estimates suggest that over 7.3 million children live in flood-prone areas (Bustamante, 2025), highlighting how caregiving burdens might amplify both perceived and real vulnerability. On average, respondents reported fewer than one child under 15 in their residence, with 50.5% reporting no children under 15 in their household.

*Race* is self-identified and collapsed into a binary indicator of white versus non-white. In Pernambuco, race maps closely onto spatial and infrastructural disadvantage, as Black and Brown Brazilians are disproportionately concentrated in precarious housing and flood-prone neighborhoods (Carvalho & Netto, 2023; Ribeiro & Carvalhaes, 2024). Religion is operationalized categorically as Catholic (35.9%), Pentecostal Evangelical (19.2%), Non-Pentecostal Evangelical (18.3%), Other (7.6%), and Atheist/Agnostic (19.0%). In peripheral Brazilian cities, Pentecostal churches often act as anchors of informal social support, though their role in mediating disaster vulnerability remains debated (Mariz, 1992; Sun et al., 2018).

*Respondent’s Mother’s Education* serves as a measure of intergenerational status and long-term positioning, shaping access to information, preparedness, and recovery resources. In northeastern Brazil, maternal education is highly stratified by race and class (Bradbury et al., 2015), and is operationalized in three categories: less than high school (44.4%), completed high school (36.3%), and some college or higher (19.2%). *Respondent’s Education* complements maternal education by capturing attained human capital and adaptive capacity in adulthood, divided into high school or less (57.9%) versus some college or more (42.1%). As in other settings, education in Pernambuco correlates strongly with access to institutional resources and the ability to mobilize support during crises. *Household Income* is reported relative to the Brazilian minimum wage (MW) and categorized into five brackets: ≤ 0.5 MW (9.5%), > 0.5–1 MW (21.0%), > 1–2 MW (25.3%), > 2–3 MW (17.8%), and > 3 MW (26.3%), representing access to flexible resources that buffer against shocks. *Employment Status* is categorized as formal employment (50.4%), informal employment (18.6%), or out of the labor force (31.1%); Brazil’s large informal sector is characterized by weak labor protections and limited access to benefits, making employment type a key dimension of vulnerability during crises.

*Water Supply Outage Frequency* is used as a proxy for infrastructural embeddedness. Measured prior to the May 2022 floods, it captures how often respondents experienced hygiene-related water outages in the prior month: none (77.9%) or at least one outage per week (22.1%). In Pernambuco, frequent outages reflect marginalization from formal municipal services and are often concentrated in informal settlements (Coleman et al., 2020). This measure thus captures infrastructural vulnerability that can compound the effects of flooding.

### Analytical approach

Building on the conceptual framework presented above, our empirical strategy integrates meteorological, spatial, and individual-level data to investigate how socioeconomic inequalities at both the individual and spatial levels shape exposure to extreme precipitation and the severity of reported impacts following the May 2022 floods in Pernambuco. We first examine whether the same dimensions of social stratification, including race, religion, income, education, number of children, and occupation, structure both measured exposure and self-reported impact. We then assess whether these disparities persist after accounting for municipality-level fixed effects and hazard exposures. To further probe the role of socio-spatial segregation, we analyze whether the fixed effects derived from the precipitation exposure model itself vary systematically by socioeconomic status, providing evidence on whether location-based hazard exposures are socially patterned across cities. Next, we test whether disadvantaged groups report more sensitivity to abnormally high precipitation, such that equivalent increases in exposures result in disproportionately higher reported impacts. Finally, we evaluate whether variation in household infrastructure, measured through piped-water connectivity, helps explain differences in flood experiences among households with similar levels of exposures.

#### Modeling stratification in exposures and impacts

We first estimate whether exposures to anomalous precipitation and reported flood impacts share similar socioeconomic gradients. Two ordinary-least-squares (OLS) regressions are estimated, one with *Anomalous Precipitation* (hazard–exposure) and one with the *Reported Impact Score* (impact) as dependent variables:

1 $$\begin{array}{c}Y_i={\beta\:}_0+\:{\beta\:}_1.Age_i+{\beta\:}_2.Race_i+{\beta\:}_3.Religion_i+{\beta\:}_4.MEduc_i+{\beta\:}_5.REduc_i+\\{\beta\:}_6.LOccu_i+\:{\beta\:}_7.NumChild_i+{\beta\:}_8.HInc_i+\varepsilon_i\end{array}$$

where $\:{Y}_{i}$ is the exposure of respondent $\:i$ to anomalous precipitation as measured by CHIRPS. The independent variables include individual and household-level socio-economic characteristics: $\:Ag{e}_{i}$ (respondent’s age), $\:Rac{e}_{i}$ (racial category), $\:Religio{n}_{i}$ (religious affiliation), $\:MEdu{c}_{i}$ (mother’s education), $\:REdu{c}_{i}$ (respondent’s education), $\:LOcc{u}_{i}$ (employment status), $\:NumChil{d}_{i}$ (number of children in the household), and $\:HIn{c}_{i}$ (household income). Finally, $\varepsilon_i$ represents an idiosyncratic error term due, e.g., to random measurement error in the dependent variable.

#### Spatial heterogeneity and sensitivity gradients

To account for residential clustering and unobserved heterogeneity across Pernambuco’s cities, we introduce municipality-level fixed effects and elevation in Eq. 2:

2 $$\begin{array}{c}Y_i={\beta\:}_0+\:{\beta\:}_1.Age_i+{\beta\:}_2.Race_i+{\beta\:}_3.Religion_i+{\beta\:}_4.MEduc_i+{\beta\:}_5.REduc_i+\\{\beta\:}_6.LOccu_i+\:{\beta\:}_7.NumChild_i+{\beta\:}_8.HInc_i+{\beta\:}_9.Elev_i+{\alpha\:}_m+\varepsilon_i\end{array}$$

where $\:Ele{v}_{i}$ is the elevation (AWS DEM) measured at the individual level. $\:{\alpha\:}_{m}$ represents municipal fixed effects and $\varepsilon_i$ is the error term, reflecting residual idiosyncratic variation and measurement error in the dependent variables after accounting for municipal fixed effects. Comparing the baseline and fixed-effects specifications reveals whether inequalities in exposure and impact are primarily people-based or place-based.

Next, we extend this model for the case in which the dependent variable is the *Reported Impact Score* to incorporate more environmental factors, anomalous precipitation intensity, and soil moisture, to adjust further for the physical context of flooding:

3 $$\begin{array}{c}Y_i={\beta\:}_0+\:{\beta\:}_1.Age_i+{\beta\:}_2.Race_i+{\beta\:}_3.Religion_i+{\beta\:}_4.MEduc_i+{\beta\:}_5.REduc_i+{\beta\:}_6.LOccu_i+\\{\beta\:}_7.NumChild_i+{\beta\:}_8.HInc_i+{\beta\:}_9.Elev_i+{\beta\:}_{10}.\:Precip_i+{\beta\:}_{11}SoilMoist_i+{\alpha\:}_m+\varepsilon_i\end{array}$$

where $\:{Y}_{i}$ represents reported impact scores, $\:Preci{p}_{i}$ is CHIRPS-based precipitation anomaly (May 2022), and $\:SoilMois{t}_{i}$ is soil moisture (ERA5-Land). Land-use is excluded from the regressions due to the sample’s uniform urban composition (over 95% urban). Elevation and soil moisture thus serve dual purposes: complementing precipitation as physical determinants of flooding and enabling tests of spatial segregation by comparing local hydrological exposure to social position. All models initially assume independently and identically distributed (i.i.d.) errors, reflecting residual idiosyncratic variation and measurement error in the dependent variables, as described above. Municipal fixed effects are subsequently included to absorb unobserved spatial heterogeneity, allowing us to isolate location-specific differences in hazard exposures.

Fixed effects estimated from the precipitation model $\:{\alpha\:}_{c}$ capture municipal-level heterogeneity in exposure to extreme rainfall after controlling for individual and household characteristics. To assess whether this heterogeneity reflects underlying patterns of social stratification, we regress the estimated municipal fixed effects on respondents’ individual socioeconomic characteristics. This approach allows us to evaluate whether individuals with particular social profiles, such as lower income, limited education, or informal labor status, are more likely to reside in cities characterized by higher exposure to anomalous rainfall, lower elevation, or greater soil saturation. In doing so, we link the spatial clustering of hazard exposure to individual-level social position, testing whether the geography of flood risk in Pernambuco is itself socially produced rather than purely environmental:

4 $$\begin{array}{c}{\alpha\:}_m={\gamma\:}_0+\:{\gamma\:}_1.Age_i+{\gamma\:}_2.Race_i+{\gamma\:}_3.Religion_i+{\gamma\:}_4.MEduc_i+{\gamma\:}_5.REduc_i+{\gamma\:}_6.LOccu_i+\\{\gamma\:}_7.NumChild_i+{\gamma\:}_8.HInc_i+{\gamma\:}_9.Elev_i+\:\varepsilon_i\end{array}$$

where $\:{\alpha\:}_{c}$ denotes the municipal-level heterogeneity (i.e., fixed effects) from the precipitation model for the municipal in which respondent *i* resides. All covariates are defined as in previous models. A significant association between individual socioeconomic characteristics and $\:{\alpha\:}_{c}$ would indicate that the spatial clustering of extreme rainfall exposure is in part socially patterned—suggesting that individuals with fewer socioeconomic resources are more likely to live in cities characterized by higher exposure to hazard conditions. We also estimate Eq. (4) using municipal fixed effects from the soil-moisture and elevation models as alternative outcome variables, providing additional insight into the socio-spatial organization of environmental vulnerability across Pernambuco.

To further assess whether disadvantaged groups exhibit stronger dose–response relationships between exposure and impact, we extend the precipitation-adjusted models by interacting anomalous rainfall with key socioeconomic characteristics such as income, education, and occupation. These interaction terms allow us to evaluate whether the marginal associations of precipitation on reported flood impact varies across social groups, thereby identifying which populations experience disproportionately higher harm as rainfall intensifies. Predicted marginal effects with 95% confidence intervals are plotted to visualize these sensitivity gradients.

Finally, we conduct several robustness checks, presented throughout Supplementary Information S.2. First, we re-estimate models using a 6-day rainfall anomaly (May 25–30, 2022) to ensure that findings are not driven by monthly aggregation and to reflect the most-intense period of precipitation. Second, we examine soil moisture as an independent outcome variable to evaluate whether its distribution across households mirrors the spatial and social gradients observed in the rainfall-anomaly exposure specifications.

#### Mediation through infrastructural embeddeness

To assess whether infrastructure disruptions help explain disparities in reported flood impacts across social groups, we estimate a mediation model in which social-economic characteristics predict both the likelihood of experiencing a service outage and the reported severity of flood-related impacts. This framework allows us to evaluate whether part of the effect of social inequality on flood outcomes operates indirectly through disruptions in basic services.

We use a binary indicator of water-supply outages, which captures the experience of at least one water-supply disruption compared to uninterrupted service, as a proxy for infrastructure vulnerability. While this measure does not fully capture household-level infrastructure embeddedness or broader dimensions of municipal-service quality, it serves as a practical proxy for how deeply a household is connected to public systems. As such, we do not expect it to fully mediate the relationship between social-structural disadvantages (e.g., income) and flood impacts. Instead, our aim is to explore whether, and to what extent, this measure can offer some insight on a household’s access to municipality services across different socioeconomic groups. 

**Fig. 2:**
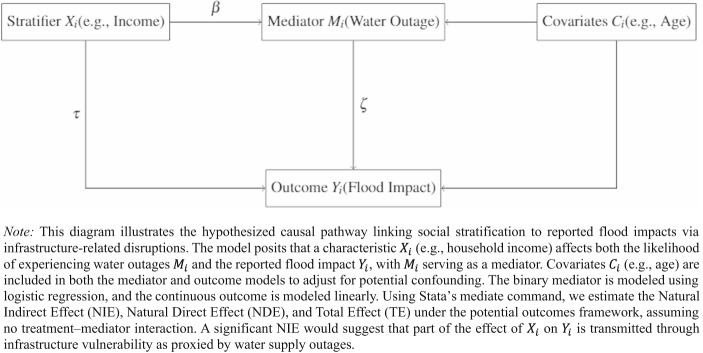
Conceptual model of mediation via water outages

Figure 2 illustrates the hypothesized causal structure underlying our mediation analysis. Building on this framework, we employ causal mediation analysis within the potential outcomes framework (Imai et al., 2010) to decompose the total effect of a social characteristic (e.g., household income) on the Reported Impact Score into the Natural Direct Effect (NDE), the Natural Indirect Effect (NIE), and the Total Effect (TE). The analysis proceeds through two linked models. First, a logistic mediator model estimates the probability that respondent *i* experienced water supply outages as a function of their social characteristics and covariates:

5 $$\begin{array}{cc}M_i=\alpha\:+\beta'.X_i+\gamma\:'.C_i+\epsilon_i&\mathrm{Mediator}\;\mathrm{model}:\;\mathrm{Logit}\end{array}$$

Second, a linear outcome model regresses the Reported Impact Score on both the characteristic and the mediator:

6 $$\begin{array}{cc}Y_i=\theta\:+\zeta\:.M_i+\tau'\:.X_i+\delta\:'\:.C_i+v_i&\mathrm{Outcome}\;\mathrm{model}:\:\mathrm{Linear}\end{array}$$

In both models, $\:X$ represents the same vector of social characteristics (e.g., household income) serving as the treatment, and $\:C$ represents the same vector of observed covariates (e.g., age) included in both models to adjust for potential confounding. The mediator model (5) estimates how social position shapes exposure to infrastructure disruptions, while the outcome model (6) isolates how much of the characteristics’ effect on flood impact operates through that disruption pathway versus directly. The terms $\:{\beta'}$, $\:{\gamma'}$, $\:{\tau'}$, and $\:{\delta'}$ represent vectors of coefficients corresponding to categorical characteristics and covariates.

These estimates reflect the average causal mediation framework, where the total effect of a social characteristic on flood impact is decomposed into the indirect effect transmitted through water supply outages (NIE) and the remaining direct effect (NDE). Estimates are computed assuming no treatment–mediator interaction, as specified in Eq. (6), and standard errors are calculated using robust sandwich estimators.

We apply this approach iteratively to all characteristics that were significant in our precipitation-adjusted fixed-effects model. A consistently significant NIE across multiple characteristics would suggest that infrastructure vulnerability operates as a general mechanism linking social stratification to flood impact. By contrast, if the NIE is significant only for specific characteristics, such as household income but not education or race, this would indicate that the mediation pathway is subgroup-specific rather than universal. Additionally, we conduct sensitivity and robustness checks using structural equation modeling (SEM) estimated via maximum likelihood and interaction-based tests to validate findings for any characteristic identified as a significant mediator.

## Results

Patterns of Stratification in Exposure and Impact.

Our first question examines whether exposures to extreme precipitation and reported-flood impacts follow similar patterns of social stratification. Table 2 presents the OLS results for both of these outcome variables from Eq. (1).

**Table 2:** OLS regression estimates of precipitation exposure and reported flood impact, including demographic, socioeconomic, and household characteristics

	(1)		(2)	
	Precipitation Exposure		Impact Score	
Age (2022)	−0.427		0.002	
	(0.301)		(0.009)	
Race (Baseline: White)				
Non-White	9.165	**	0.174	
	(2.962)		(0.090)	
Religion (Baseline: Catholic)				
Non-Pentecostal Evangelical	2.176		0.105	
	(3.751)		(0.114)	
Pentecostal Evangelical	8.937	*	0.182	
	(3.749)		(0.114)	
Other religion	19.076	***	0.098	
	(5.322)		(0.162)	
Atheist/Agnostic	6.364		0.012	
	(3.732)		(0.113)	
Respondent’s Mother’s Education (Baseline: Less than High School)				
Highschool	10.284	***	0.096	
	(3.056)		(0.093)	
Some College or More	5.847		0.233	
	(4.009)		(0.122)	
Respondent’s Education (Baseline: High School or Less)				
Some College or More	−0.614		0.039	
	(3.091)		(0.094)	
Employment Status (Baseline: Formal Employment)				
Informal Employment	3.879		0.322	**
	(3.762)		(0.114)	
Out of the Labor Force	5.018		0.088	
	(3.294)		(0.100)	
Current Number of Children < 15	−1.043		0.131	**
	(1.606)		(0.049)	
Household Income (Baseline: 3 Minimum Wage or Above)				
1/2 Minimum Wage or Below	−13.140	*	0.573	**
	(5.717)		(0.174)	
1/2 to 1 Minimum Wage	−5.683		0.654	***
	(4.440)		(0.135)	
1 to 2 Minimum Wage	−0.533		0.526	***
	(3.973)		(0.121)	
2 to 3 Minimum Wage	−3.279		0.196	
	(4.095)		(0.124)	
Intercept	242.346	***	−0.882	**
	(10.196)		(0.310)	
Number of observations	1411		1411	
F statistic	3.3984		5.3431	
R-squared	0.0375		0.0578	

^***^ *p <* 0.001, ^**^ *p <* 0.01, ^*^ *p <* 0.05. The two models presented above are based on data from the restricted-access version of the DZC survey. Precipitation exposure has a mean of 244.034 mm and an SD of 49.9 mm with a range of 24.17 mm to 332.76 mm. Weighted impact score has a mean of 0.89 with a range of 0 to 5

In the precipitation hazard-exposure model (Column 1), *Age* is not significant, *non-white* respondents experience 9.17 mm higher exposure than whites (*p* < 0.001), *Pentecostal Evangelicals* and those of *Other Religions* have greater exposure than Catholics at + 8.94 mm (*p* < 0.05) and + 19.08 mm (*p* < 0.001), respectively. Respondents whose mothers achieved high-school education faced + 10.28 mm (*p* < 0.001) larger precipitation anomalies compared to respondents with mothers with less-than-high-school counterparts and *Respondent’s Education*, *Employment Status*, and *Number of Children* are not significant. Regarding *Household Income*, those earning 0.5 the minimum wage or below were exposed to 13.14 mm (*p* < 0.05) less rain than their high-earning counterparts earning 3 times the minimum wage or more.

In the *Reported Impact* model (Column 2), individuals engaged in *Informal Employment* report impact scores approximately 0.32 points higher (*p* < 0.01) than their formally employed counterparts, the *Number of Children Under 15* within the household is significantly associated with reported impact, with each additional child corresponding to a 0.15-point increase (*p* < 0.01) and a clear income gradient is evident: compared to respondents earning > 3 MW, those earning > 1–2 MW, > 0.5–1 MW, and ≤ 0.5 MW report substantially higher impact scores—by 0.53 (*p* < 0.001), 0.65 (*p* < 0.001), and 0.58 (*p* < 0.01) points, respectively—highlighting the strong socioeconomic gradient in reported flood disruption. Both model fits are modest, with relatively low R^2^ values^7^.

### Adjusting for Spatial Heterogeneity and Hazards

Introducing municipal fixed effects (Eq. 2) substantially reduces the number of significant predictors in the exposure model. Table 3, Column 1, presents the results of the exposure model in which none of the predictors are significant, even with the inclusion of elevation as a covariate.

**Table 3:** Fixed-effects regression estimates of precipitation exposure and reported flood impact, with and without precipitation adjustment, including demographic, socioeconomic, and household characteristics

	(1)		(2)		(3)	
	Precipitation Exposure		Impact Score (No Climate)		Impact Score (Climate)	
Age (2022)	−0.123		0.000		−0.001	
	(0.171)		(0.009)		(0.009)	
Race (Baseline: White)						
Non-White	2.352		0.106		0.109	
	(1.677)		(0.093)		(0.093)	
Religion (Baseline: Catholic)						
Non-Pentecostal Evangelical	−3.593		0.021		0.019	
	(2.116)		(0.117)		(0.117)	
Pentecostal Evangelical	1.961		0.127		0.130	
	(2.145)		(0.118)		(0.118)	
Other religion	2.088		0.017		0.018	
	(2.958)		(0.163)		(0.163)	
Atheist/Agnostic	1.917		−0.021		−0.020	
	(2.118)		(0.117)		(0.117)	
Respondent’s Mother’s Education (Baseline: Less than High School)						
Highschool	−0.750		−0.006		−0.006	
	(1.740)		(0.096)		(0.096)	
Some College or More	−1.193		0.079		0.077	
	(2.260)		(0.125)		(0.125)	
Respondent’s Education (Baseline: High School or Less)						
Some College or More	1.876		0.092		0.093	
	(1.742)		(0.096)		(0.096)	
Employment Status (Baseline: Formal Employment)						
Informal Employment	−3.399		0.255	*	0.250	*
	(2.118)		(0.117)		(0.117)	
Out of the Labor Force	−0.068		0.052		0.052	
	(1.873)		(0.103)		(0.103)	
Current Number of Children < 15	−1.056		0.138	**	0.137	**
	(0.910)		(0.050)		(0.050)	
Household Income (Baseline: 3 Minimum Wage or Above)						
1/2 Minimum Wage or Below	2.570		0.722	***	0.726	***
	(3.277)		(0.181)		(0.181)	
1/2 to 1 Minimum Wage	−1.226		0.653	***	0.648	***
	(2.526)		(0.139)		(0.140)	
1 to 2 Minimum Wage	0.521		0.540	***	0.539	***
	(2.246)		(0.124)		(0.124)	
2 to 3 Minimum Wage	1.694		0.257	*	0.258	*
	(2.310)		(0.127)		(0.128)	
elevation	−0.012		−0.002		−0.002	
	(0.024)		(0.001)		(0.001)	
Anomaly Precipitation (mm)					−0.001	
					(0.002)	
Soil Moisture (m)					0.001	
					(0.001)	
Intercept	249.396	***	−0.562		−0.531	
	(5.955)		(0.329)		(0.735)	
Number of observations	1358		1358		1358	
F statistic	1.1525		4.6921		4.2361	
R-squared	0.6958		0.1305		0.13105	
Total number of absorbed categories	47		47		47	

^***^ *p <* 0.001, ^**^ *p <* 0.01, ^*^ *p <* 0.05. The three models presented above are based on data from the restricted-access version of the DZC survey. Precipitation exposure has a mean of 244.03 4 mm and an SD of 49.9 mm with a range of 24.17 mm to 332.76 mm. Weighted impact score has a mean of 0.89 with a range of 0 to 5. The fixed effects specifications exclude municipalities with insufficient within-group variation, primarily those represented by a single respondent; the analytic sample for fixed effects models is therefore slightly smaller (*n* = 1,358) than for OLS (*n* = 1,411)

In contrast, the model of reported flood impact without precipitation adjustment (Column 2) reveals persistent disparities beyond those captured by the municipal fixed effects. Respondents working in *Informal Employment* report significantly higher impact scores compared to those in formal employment (*p* < 0.05), *Household Income* and the *Current Number of Children Under 15* remain strong and significant predictors. Each additional child is associated with a 0.14-point increase in reported impact. Lower-income households report substantially higher flood impacts, with effect sizes increasing in magnitude as income decreases—from 0.337 points among those earning one to two MWs to 0.457 points among those earning ≤ 0.5 MW (*p* < 0.001).

The fully adjusted model for the Reported Impact Score (Column 3), which includes all hazard-exposure variables, namely, anomalous precipitation, soil moisture, and elevation, as well as municipal-level fixed effects, is a direct test of whether disparities in reported flood impact reflect differences in measured exposures. Notably, none of the proxies of hazard exposure themselves have statistically significant coefficient estimates, yet *Employment Type*, *Household Income*, and *Number of Children Under 15* remain significantly associated with impact scores.

Given the attenuation of socioeconomic effects on hazard exposure in Table 3, Column 1, but their persistence in the impact model, we further examine whether the remaining spatial heterogeneity captured patterns of socioeconomic segregation. To do so, we regressed the estimated municipal-level fixed effects from the precipitation exposure model, as well as those from the elevation and soil moisture models (SI S.2.5), on respondents’ socioeconomic characteristics. These regressions (Eq. 4), evaluate whether unobserved municipal-level variations in environmental exposure are systematically associated with the social composition of residents. Results are shown in Table 4 below.

**Table 4:** Regression estimates of fixed effect coefficients on demographic, socioeconomic, and household characteristics

	(1)		(2)		(3)	
	Precipitation		Elevation		Soil Moisture	
Age (2022)	−0.199		−0.108		0.798	
	(0.243)		(0.691)		(0.414)	
Race (Baseline: White)						
Non-White	5.623	*	−17.759	**	5.525	
	(2.392)		(6.795)		(4.076)	
Religion (Baseline: Catholic)						
Non-Pentecostal Evangelical	3.283		−10.186		11.417	*
	(3.016)		(8.568)		(5.140)	
Pentecostal Evangelical	4.734		−19.106	*	9.549	
	(3.015)		(8.566)		(5.139)	
Other religion	13.247	**	−33.378	**	0.388	
	(4.254)		(12.085)		(7.250)	
Atheist/Agnostic	1.799		−4.413		4.014	
	(3.020)		(8.580)		(5.147)	
Respondent’s Mother’s Education (Baseline: Less than High School)						
Highschool	9.537	***	−24.808	***	3.277	
	(2.460)		(6.990)		(4.193)	
Some College or More	6.433	*	−19.692	*	1.973	
	(3.224)		(9.160)		(5.495)	
Respondent’s Education (Baseline: High School or Less)						
Some College or More	−2.059		10.458		3.020	
	(2.488)		(7.070)		(4.241)	
Employment Status (Baseline: Formal Employment)						
Informal Employment	8.153	**	−18.503	*	2.595	
	(3.021)		(8.584)		(5.149)	
Out of the Labor Force	5.673	*	−21.006	**	5.083	
	(2.656)		(7.547)		(4.527)	
Current Number of Children < 15	−0.575		4.117		−5.498	*
	(1.294)		(3.676)		(2.205)	
Household Income (Baseline: 3 Minimum Wage or Above)						
1/2 Minimum Wage or Below	−12.797	**	32.423	*	−8.974	
	(4.647)		(13.201)		(7.919)	
1/2 to 1 Minimum Wage	−4.482		16.983		6.246	
	(3.561)		(10.117)		(6.069)	
1 to 2 Minimum Wage	−1.765		9.722		6.317	
	(3.196)		(9.081)		(5.447)	
2 to 3 Minimum Wage	−4.452		15.761		−2.691	
	(3.297)		(9.366)		(5.618)	
Intercept	−4.449		28.435		−34.472	*
	(8.249)		(23.436)		(14.059)	
Number of observations	1358		1358		1358	
F statistic	3.3124		2.9836		1.5501	
R-squared	0.0380		0.0344		0.0182	

*** *p* < 0.001, ** *p* < 0.01, * *p* < 0.05. Each column reports OLS coefficients (SE in parentheses) from regressions of the city fixed effects coefficients on socioeconomic characteristics as described by Eq. (4). The dependent variable is the city FE from (1) the precipitation-anomaly model, (2) the elevation model, and (3) the soil-moisture model. Positive coefficients indicate a higher likelihood of residing in municipalities with higher-than-average values of the column’s outcome—i.e., (1) larger precipitation anomalies, (2) higher elevation, or (3) wetter antecedent soils (greater saturation). Units inherit from the underlying models (precipitation in mm, elevation in m, soil moisture in m^3^)

In the fixed-effect regression, where precipitation is the outcome, (Column 1), several indicators are statistically significant. Individuals identifying as *non-White* are more likely to reside in cities with higher exposure to extreme rainfall of + 5.62, (*p* < 0.05). Respondents in *Other Religions* show similarly elevated likelihood of residence in higher- exposure cities + 13.25, (*p* < 0.01). Higher *maternal education* corresponds to a greater likelihood of exposure, with those whose mothers completed high school or some college showing coefficients of + 9.54 (*p* < 0.001) and + 6.43 (*p* < 0.05), respectively. Labor characteristics are also significant. Individuals in *Informal Employment* or *Out of the Labor Market* are more likely to live in municipalities with higher precipitation + 8.15 and + 5.67, respectively. In contrast, lower-income groups, particularly those earning half the minimum wage or less, have a lesser likelihood of living in cities with high exposure − 12.80, (*p* < 0.01).

The elevation fixed-effect model shows the same characteristics to be significant, albeit with an inverted spatial pattern. Individuals identifying as *non-White* and those in *Pentecostal* or *Other Religions* have higher likelihoods of residing in lower-elevation areas − 17.76, −19.11, and − 33.38, respectively. Respondents with more-educated mothers are less likely to live in higher-elevation areas, as indicated by negative coefficients for *Maternal High School* − 24.81, (*p* < 0.001) and *College or More* − 19.69, (*p* < 0.05). Those with *Informal Employment* and *Out of the Labor Force* also show negative and significant coefficients − 18.5 and − 21.01, consistent with concentration in lower-lying terrain. Conversely, the lowest-income group exhibits a positive association + 32.42, (*p* < 0.05), suggesting that some low-income respondents are more likely to reside in higher-elevation municipalities that experience limited rainfall anomalies.

Results from the soil-moisture residual regression are weaker overall, with few statistically significant associations. Households with more *Children Under 15* are more likely to reside in slightly drier areas, with a coefficient of −5.50, (*p* < 0.05). Similarly, those of the non-Pentecostal evangelical faith show higher likelihood of being in areas with high soil moisture at + 11.42 (*p* < 0.05). Across models, the relatively low R^2^ values (ranging from 0.02 to 0.04) indicate that although socioeconomic characteristics contribute modestly to spatial variation in hazard contexts, a large share of the variance remains unexplained by our observed variables.

**Fig. 3:**
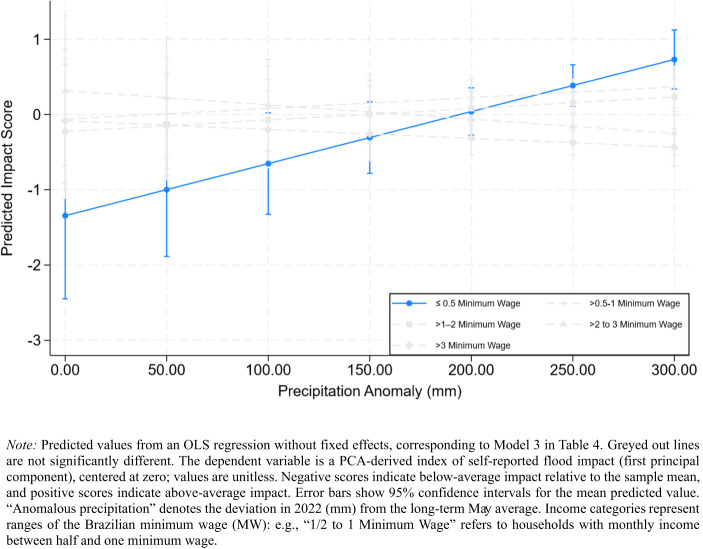
*Estimated Self-Reported Impact Scores by Anomalous Precipitation and Household Income*

**Fig. 4:**
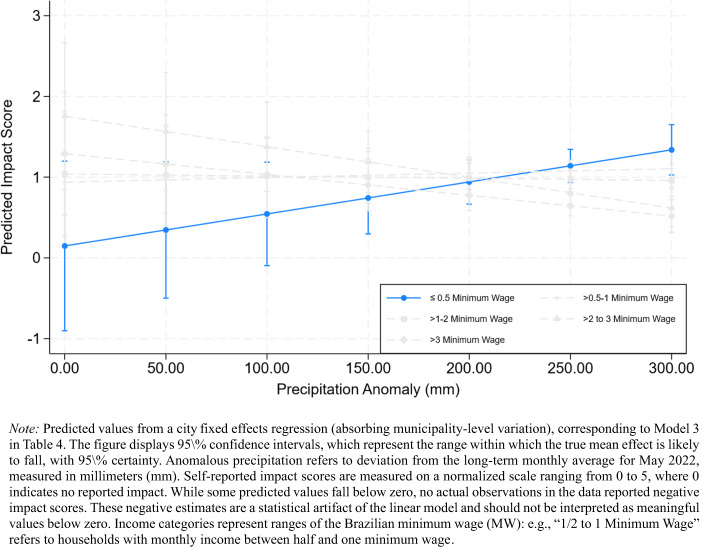
*Estimated Self-Reported Impact Scores by Anomalous Precipitation and Household Income (Fixed Effects Model)*

To assess whether disadvantaged groups exhibit stronger dose–response relationships between precipitation exposure and reported flood impacts, we interacted *Anomalous Precipitation* with key socioeconomic characteristics. Among all tested interactions (See SI S.1 for all combinations), only the interaction between *Household Income* and *Anomalous Precipitation* is statistically significant, and this relationship remains robust after introducing municipal-level fixed effects. The corresponding marginal effects are shown in Figs. 3 and 4.

Figure 3 presents the unadjusted marginal effects of precipitation across income categories. The slope is significantly steeper for the lowest-income households, those earning ≤ 0.5 MW, indicating that small increases in rainfall correspond to disproportionately higher reported-flood impacts in this group (*p* < 0.05). Middle- and upper-income households, in contrast, display relatively flat slopes, suggesting limited change in perceived flood disruption as precipitation increases. Otherwise, these groups are not significantly different than the highest income group.

Figure 4 incorporates municipal-level fixed effects to control for spatial clustering. The income–precipitation interaction for the lowest-income group remains positive and significant, though its magnitude is attenuated, confirming that socioeconomic sensitivity to precipitation persists beyond locational differences in exposure. The other income categories remain statistically indistinguishable from their reference category, indicating that the dose–response relationship is concentrated among the poorest respondents.

### Household Water Supply Disruptions as a Mediator of Income Disparities in Flood Impact

To examine whether disruptions in household water access mediate the relationship between income and reported flood impact, we estimate the causal-mediation model (Eqs. 5 & 6), using maximum likelihood with robust standard errors. Table 5 reports the NIE, NDE, and TE for each income group relative to the highest-income category (three minimum wages or more). The binary mediator captures whether a respondent experienced at-least-one water-supply interruption prior to the flood.

**Table 5:** Causal mediation estimates of income effects on reported flood impact, mediated by water outages

Income Group (Ref: 3 Minimum Wage or More)	NIE	NDE	Total Effect	Proportion Mediated
1/2 Minimum Wage or Below	0.082^***^	0.599^***^	0.680^***^	12.0%
	(0.029)	(0.196)	(0.196)	
1/2 to 1 Minimum Wage	0.080^***^	0.624^***^	0.704^***^	11.4%
	(0.025)	(0.129)	(0.130)	
1 to 2 Minimum Wage	0.055^***^	0.491^***^	0.546^***^	10.0%
	(0.019)	(0.109)	(0.110)	
2 to 3 Minimum Wage	0.043^*^	0.131	0.174	24.5%
	(0.017)	(0.102)	(0.100)	

Outcome model = linear; mediator model = logit. Mediator is any pre-flood water-supply outage (≥ 1 vs. none). Treatment is multivalued household income (four categories) relative to ≥ 3 minimum wages (MW). NIE = Natural Indirect Effect; NDE = Natural Direct Effect; Total Effect = NIE + NDE. Proportion mediated = NIE/Total Effect (from point estimates; no SEs). Robust SEs in parentheses. Covariates in both equations: age, race, religion, mother’s education, respondent’s education, and employment status. Outcome equation does not include a treatment–mediator interaction. Mediation effects were estimated under the potential outcomes framework (Imai et al., 2010) using Stata’s mediate command, which decomposes the total effect into the NIE (effect transmitted through water-supply outages) and the NDE (remaining direct effect), assuming sequential ignorability of the mediator. Significance: * *p* < 0.05, ** *p* < 0.01, *** *p* < 0.001

Across income groups, the results indicate statistically significant but partial mediation through household water supply. The proportion of the total effect mediated (NIE)/(TE) ranges from approximately 12% to 25%. Specifically, mediation accounts for about 12% of the total effect among households earning ≤ 0.5 MW, 11% for those earning > 0.5–1 MW, and 10% for those earning > 1–2 MW. For the > 2–3 MW group, the proportion mediated is higher at roughly 24%.^8^

## Discussion and conclusion

Three patterns stand out in this investigation of how socioeconomic inequalities, at both individual and spatial scales, shape exposure and impact. First, once accounting for municipality characteristics, socioeconomic gradients in *measured hazard exposure* largely attenuate, whereas gradients in *reported impact* remain. Second, social structures influence residential sorting across municipalities and by elevation, layering risk onto those already disadvantaged: people with preexisting vulnerabilities are more likely to live in high-risk areas and, in turn, to bear greater losses along lines of economic vulnerability, such as income. Third, infrastructural disenfranchisement—proxied by lack of piped municipal water—mediates a non-trivial share of the income–impact relationship. Reading the five dimensions of DRR research through a sociological lens reveals layered vulnerabilities that not only increase the likelihood of exposure based on where people live but also intensify the severity of harm once floods occur.

Our findings show some bivariate social patterning of anomalous rainfall, but those associations vanish once municipal fixed effects are introduced. This is consistent with a ‘place effect’ on exposure: the spatial clustering of the May 2022 rainfall anomaly operates primarily at the municipal scale rather than through who people are. The impact models tell a more complex story. While municipal fixed effects significantly improve model fit, robust socioeconomic gradients persist after their inclusion — lower income, informal employment, and larger numbers of children are associated with higher disruption even after adjusting for municipal context and physical conditions (i.e., precipitation anomaly, elevation, soil moisture). This suggests that place and social position are both implicated in flood impacts: municipality-level factors account for baseline variation in disruption, while individual socioeconomic characteristics are associated with who within those places experiences the greatest burden. In DRR research terms, this decoupling between hazard/exposure and impact aligns with sociological accounts that vulnerability is generated by unequal access to “flexible resources” (Link & Phelan, 1995) and with evidence that impacts can remain unequal when hazard is held constant (Torche & Nobles, 2024). The results thus reinforce the idea that the transition from hazard to harm is importantly socially mediated rather than mechanically determined by rainfall totals.

To probe the social production of space (Raker, 2025), we regress the municipal fixed effects from the precipitation model on individual SES characteristics. Non-white identity, non-Catholic affiliations (especially “other” minority religions), higher maternal education, and labor market precarity are associated with higher likelihood of residing in high-exposure cities, whereas the very-lowest-income group are less likely to reside in high-exposure cities. For the elevation municipal fixed effects, signs flip for several markers (e.g., non-white and Pentecostal/other religions are associated with lower elevation). Taken together, these patterns suggest distinct pathways to risk: some groups are overrepresented in municipalities receiving more anomalous rainfall, while others (or the same groups) are concentrated in lower-lying terrain that heightens runoff and inundation risks. The fact that municipal fixed effects from the precipitation and elevation models diverge for several social groups underscores that rainfall intensity and hydrological susceptibility operate as distinct spatial risks at the municipal level, justifying our decision to include elevation and soil moisture alongside rainfall in the impact models.

Interactions between precipitation and income show that the marginal effect of additional rain is concentrated among the poorest households. Even after absorbing municipal-level heterogeneity, the lowest-income group exhibits a steeper slope relating rain to impact, consistent with the notion of stratified sensitivity (Torche & Nobles, 2024). However, this finding must be tempered with the knowledge that the pattern existed only for those who reported being among the lowest incomes, not a universal pattern across socioeconomic groups.

Causal-mediation analysis indicates that pre-event water service interruptions account for about 10–12% of the income–impact association for lower and lower-middle income groups and roughly 24% for households with incomes of two to three times the minimum wage. These proportions are modest but systematic, in keeping with evidence that infrastructural embeddedness structures disaster experiences in highly unequal urban settings. They also indicate that the bulk of the gradient remains direct, which is consistent with other unmeasured channels (e.g., housing quality, informal tenure, savings and credit, caregiving burdens) highlighted in the disaster–inequality literature.

Several considerations qualify these conclusions. First, our sample is constrained to reports from women ages 18 to 34 in 2020; thus, we cannot expand conclusions to the elderly, men, or younger people, all of whom might experience flash floods differently. Second, the survey has a nontrivial sample attrition of 30% between the 2nd and 3rd waves because the baseline was implemented during Covid-19 lockdown, though we apply weights to attempt to correct for those losses. Third, CHIRPS provides macro-level estimations of rainfall gridded at 4.7 km^2^ and may not capture micro-convective cells, potentially leading to attenuation of sub-kilometer variations and introducing attenuation-type errors, a common issue of satellite-based analysis. Fourth, the explanatory power of several models, R^2^ < 0.05 in specifications without municipal fixed effects, is modest, though to be expected because flash-flood impacts arise from complex interactions between physical and social processes that are only partially captured by our variables. Fixed effects at the municipal scale absorb broad spatial heterogeneity but cannot adjudicate neighborhood-scale processes or fully rule out omitted neighborhood characteristics.

*Robustness*. To assess the sensitivity of our findings, we undertook several robustness checks addressing some of these limitations. First, to validate the CHIRPS-based hazard measure, we re-estimated models using two alternative exposure metrics: (i) soil moisture from ERA5-Land and (ii) a 6-day rainfall anomaly (25–30 May 2022) for the peak-precipitation window. Both specifications yielded consistent patterns of associations, confirming the robustness of our exposure measure (SI S.2.5). Second, to evaluate the stability of the impact score, we disaggregated the principal component into its four constituent indicators, recoded each into binary impact outcomes, and re-estimated models using logistic regressions. Results were substantively similar to the composite specification, indicating that no single dimension disproportionately drove observed socioeconomic gradients (SI S.2.2). We repeated this process for the mediation analysis with similar results. Third, in response to concerns about low model fit, we re-ran the hazard-exposure models, including elevation as a covariate. This addition improved the R^2^ considerably, confirming that spatial elevation is consistent with substantial variance in rainfall anomalies; however, we retained the more parsimonious specification in the main text to isolate the predictive contribution of socioeconomic characteristics (See SI S.7). Fourth, to address potential multicollinearity among socioeconomic indicators (income, education, and employment), we computed variance inflation factors (VIF) for all models, all of which fell below the conventional threshold of 5. Importantly, to evaluate attrition bias, we tested the association between household income, age, and respondents’ education in Wave 2 and subsequent participation in Wave 3. Neither Pearson’s Chi^2^ test nor logistic regression predicting attrition indicated a statistically significant relationship (SI S.6). Finally, to assess whether municipality sampling affects our findings, we drop the three most-populous municipalities (excluding Recife) and re-estimate all models; the inferences are unchanged. We also restrict the sample to Recife alone and again obtain substantively similar results (SI S.8). Collectively, these checks increase confidence that the reported relationships are not artifacts of measurement error, model specification, or sample selection.

This study underscores that vulnerability to extreme precipitation is shaped by social inequality as well as by hydrometeorological hazards. Even after accounting for municipal-level heterogeneity, lower-income households, those with informal employment, and larger households report higher flood disruptions, indicating that socioeconomic position, not just physical exposure, is associated with who suffers most. As recent attribution studies suggest, the intensity and frequency of heavy rainfall events in northeastern Brazil are likely to increase under continued warming (Vasconcelos Junior et al., 2024); our results underscore the need for targeted adaptation policies that address the socioeconomic dimensions of flood vulnerability. To avoid unequal and possibly inequality-increasing effects, municipal and state authorities should prioritize drainage and water-supply reliability in low-income neighborhoods with high informal employment and large numbers of children, pairing infrastructure investments with: accessible early-warning communication through schools and clinics, safe evacuation routes and shelters with sanitation and childcare, continuity plans for caregiving, cash or in-kind assistance timed to care demands, and fully staffed, trained civil-defense units equipped to reach poorer areas first.

## Supplementary information

Below is the link to the electronic supplementary material.

Supplementary Material 1 (DOCX 430 KB)

## Data Availability

No datasets were generated or analysed during the current study.

## Footnotes

1 A thunderstorm in which new development takes place on the upwind side (usually the west or southwest side), such that the storm seems to remain stationary or propagate in a backward direction.

2 In this article, we use flash floods and pluvial floods interchangeably.

3 Because CHIRPS data do not account for sub-kilometer topography or urban drainage, we supplement rainfall measures with elevation estimates from Digital Elevation Models (DEM) at the individual-residence location and soil-moisture data to better capture how runoff and drainage dynamics can exacerbate flooding in the exposure models.

4 The household-level rainfall exposure is obtained by overlaying each respondent’s latitude–longitude on the 0.05° CHIRPS grid and extract the May 2022 anomalous precipitation.

5 An attrition rate of 30% between the second and third waves is noted; analysis of the randomness of attrition is conducted in the limitation and robustness sections of the discussion.

6 See robustness checks for these reports, with an unweighted index and the four items separately, in SI S.2.2.

7 For both models, overall model fits are modest but consistent with expectations given the complexity of social and environmental processes under study. The relatively low R^2^ values, 0.038 for precipitation exposure (Column 1) and 0.058 for reported impact (Column 2), indicate that the included individual- and household-level socioeconomic characteristics are consistent with only small shares of total variances of the two outcome variables.

8 Full mediation results for alternative characteristics (e.g., number of children under 15), mediation configurations, and individual component mediation analysis are presented in SI S.4.1.
