# Supplementary material for: Who is exposed and who is harmed? Social disparities in flood exposure and impact in Pernambuco, Brazil
Source: Popul Environ. 2026 Apr 21;48(2):10. doi: 10.1007/s11111-026-00523-z (PMC13099789; doi:10.1007/s11111-026-00523-z)
Supplement: Supplementary file 1 — Supplementary Material 1 (DOCX 430 KB) [file 11111_2026_523_MOESM1_ESM.docx]

**Online Supplementary Information**

Who is exposed and who is harmed? Social disparities in flood exposure and impact in Pernambuco, Brazil

## S.1 Model Configurations for Interaction Tests

Table S1. Model Configurations for Interaction Tests Between Precipitation and Socioeconomic/Demographic Variables

| 1. **Age Interaction:**   OLS Model (Eq. 1) + *(Age x Precipitation)* |
| --- |
| 1. **Race Interaction:**   OLS Model (Eq. 1) + *(White x Precipitation)* |
| 1. **Household Income Interaction:**   OLS Model (Eq. 1) + *(Less than 1/2 MW x Precipitation) + (1/2 to 1 MW x Precipitation)*  *+ (1 to 2 MW x Precipitation) + (2 to 3 MW x Precipitation)*  *+ (3 MW or Above x Precipitation)* |
| 1. **Religion Interaction:**   OLS Model (Eq. 1) *+ (Catholic x Precipitation) + (Non-Pentecostal Evangelical x Precipitation)*  *+ (Pentecostal Evangelical x Precipitation) + (Other x Precipitation)*  *+ (Atheist/Agnostic x Precipitation)* |
| 1. **Employment Status Interaction:**   OLS Model (Eq. 1) + *(Formal Employment x Precipitation)*  *+ (Informal Employment x Precipitation) + (Out of the Labor force x Precipitation)* |
| 1. **Water Outage Frequency Interaction:**   OLS Model (Eq. 1) *+ (No Outage x Precipitation) + (1-2 times x Precipitation)*  *+ (3 times or More x Precipitation)* |
| 1. **Current Number of Children Interaction:**   OLS Model (Eq. 1) *+ (Current Number of Children Under 15 x Precipitation)* |

## S.2 Sensitivity Analysis of Self-Reported Scores

### S.2.1 Principal Components Analysis of Disruption Indicators

N = 1,411; Components = 4, Rotation = unrotated (principal), Trace = 4, Rho = 1.0000

| Panel A. Eigenvalues and explained variance | | | | | | | | |
| --- | --- | --- | --- | --- | --- | --- | --- | --- |
| **Component** | **Eigenvalue** | | **Difference** | | **Proportion** | | **Cumulative** | |
| Comp 1 | 2.31619 | | 1.52475 | | 0.579 | | 0.579 | |
| Comp 2 | 0.79144 | | 0.24795 | | 0.1979 | | 0.7769 | |
| Comp 3 | 0.54349 | | 0.19461 | | 0.1359 | | 0.9128 | |
| Comp 4 | 0.34888 | | — | | 0.0872 | | 1 | |
| Panel B. Component coefficients (eigenvectors) | | | | | | | | |
| **Variable** | | **Comp 1** | | **Comp 2** | | **Comp 3** | | **Comp 4** |
| Lack of connection | | 0.4793 | | 0.4473 | | -0.7543 | | 0.0367 |
| Flooding | | 0.4543 | | 0.6064 | | 0.6507 | | 0.0499 |
| Homelessness | | 0.5213 | | -0.5117 | | 0.0609 | | 0.6803 |
| Loss of goods | | 0.5406 | | -0.4127 | | 0.0632 | | -0.7303 |

### S.2.2 Analysis of Standalone Disruption Indicators

To verify that our main conclusions are not an artifact of the PCA-based Reported Impact Score, we re-estimated the models using each disruption indicator as a standalone binary outcome. Because the original response scales were highly skewed—especially for *loss of home/becoming homeless* and *loss/damage of goods*—we dichotomized each indicator so that “not affected” = 0 and any level of affect (“slightly” to “very affected”) = 1. Table S2 summarizes the distribution of the four indicators; roughly half of respondents report at least one day of utility/connection outage, while flooding and damages are less frequent but non-negligible.

Table S2. Descriptive Statistics of Disruption Indicators

|  | Summary |
| --- | --- |
| N | 1,411 |
| Lack of water, electricity, internet, or cellular signal for at least one day |  |
| Not affected | 710 (50.3%) |
| Slightly affected | 126 (8.9%) |
| Moderately affected | 138 (9.8%) |
| Affected | 164 (11.6%) |
| Significantly affected | 85 (6.0%) |
| Very affected | 188 (13.3%) |
| Flooding at home, at your building, street, or neighborhood |  |
| Not affected | 527 (37.3%) |
| Slightly affected | 93 (6.6%) |
| Moderately affected | 143 (10.1%) |
| Affected | 194 (13.7%) |
| Significantly affected | 154 (10.9%) |
| Very affected | 300 (21.3%) |
| Losing your home or becoming homeless |  |
| Not affected | 1,229 (87.1%) |
| Slightly affected | 65 (4.6%) |
| Moderately affected | 29 (2.1%) |
| Affected | 19 (1.3%) |
| Significantly affected | 18 (1.3%) |
| Very affected | 51 (3.6%) |
| Loss of goods such as a car, motorcycle, phone or home appliances |  |
| Not affected | 1,143 (81.0%) |
| Slightly affected | 79 (5.6%) |
| Moderately affected | 35 (2.5%) |
| Affected | 46 (3.3%) |
| Significantly affected | 31 (2.2%) |
| Very affected | 77 (5.5%) |

For each indicator—(1) loss of connection, (2) flooding at/near the home, (3) loss of home/becoming homeless, and (4) loss/damage of goods—we estimated logistic regressions with the same covariate set used in the WRIS models: age, race, religion, mother’s education, respondent’s education, labor-market status (formal, informal, out of labor force), number of children under 15, household income categories (baseline: ≥3 minimum wages), and environmental controls (May 2022 precipitation anomaly in mm, elevation, and soil moisture). Robust standard errors are reported; the estimation sample is $N=1,411$ (Table S3).

Table S3. Logistic Regression Estimates of the four indicators

|  | (1) | | (2) | | (3) | | (4) | |
| --- | --- | --- | --- | --- | --- | --- | --- | --- |
|  | Loss of Connection | | Flooding | | Loss of Home/Homelessness | | Loss/Damage of Goods | |
| Age (2022) | -0.013 |  | -0.008 |  | 0.006 |  | -0.002 |  |
|  | (0.013) |  | (0.013) |  | (0.019) |  | (0.016) |  |
| Race (Baseline: White) |  |  |  |  |  |  |  |  |
| Non-White | 0.237 |  | 0.220 |  | 0.325 |  | 0.120 |  |
|  | (0.126) |  | (0.132) |  | (0.205) |  | (0.166) |  |
| Religion (Baseline: Catholic) |  |  |  |  |  |  |  |  |
| Non-Pentecostal Evangelical | -0.148 |  | 0.084 |  | -0.170 |  | -0.098 |  |
|  | (0.159) |  | (0.167) |  | (0.239) |  | (0.207) |  |
| Pentecostal Evangelical | -0.233 |  | 0.238 |  | -0.054 |  | 0.089 |  |
|  | (0.158) |  | (0.168) |  | (0.219) |  | (0.193) |  |
| Other religion | -0.140 |  | -0.072 |  | -0.144 |  | -0.125 |  |
|  | (0.224) |  | (0.232) |  | (0.358) |  | (0.306) |  |
| Atheist/Agnostic | 0.076 |  | 0.132 |  | -0.321 |  | 0.077 |  |
|  | (0.158) |  | (0.167) |  | (0.250) |  | (0.201) |  |
| Respondent’s Mother’s Education (Baseline: Less than High school) |  |  |  |  |  |  |  |  |
| Highschool | -0.079 |  | 0.137 |  | -0.225 |  | -0.200 |  |
|  | (0.130) |  | (0.136) |  | (0.190) |  | (0.164) |  |
| Some College or More | 0.229 |  | 0.264 |  | -0.007 |  | 0.044 |  |
|  | (0.171) |  | (0.181) |  | (0.271) |  | (0.222) |  |
| Respondent’s Education (Baseline: High School or Less) |  |  |  |  |  |  |  |  |
| Some College or More | 0.074 |  | 0.247 |  | 0.205 |  | 0.281 |  |
|  | (0.131) |  | (0.138) |  | (0.200) |  | (0.170) |  |
| Employment Status (Baseline: Formal Employment) |  |  |  |  |  |  |  |  |
| Informal Labor Market | 0.391 | * | 0.454 | ** | 0.355 |  | 0.104 |  |
|  | (0.159) |  | (0.174) |  | (0.224) |  | (0.194) |  |
| Out of Labor Market | -0.019 |  | -0.031 |  | 0.128 |  | -0.165 |  |
|  | (0.138) |  | (0.144) |  | (0.207) |  | (0.178) |  |
| Current Number of Children Under 15 | 0.068 |  | -0.002 |  | 0.195 | * | 0.220 | ** |
|  | (0.068) |  | (0.071) |  | (0.090) |  | (0.080) |  |
| Household Income (Baseline: 3 Minimum Wage or Above) |  |  |  |  |  |  |  |  |
| 1/2 MW or Below | 0.627 | ** | -0.191 |  | 1.378 | *** | 1.204 | *** |
|  | (0.243) |  | (0.252) |  | (0.370) |  | (0.304) |  |
| 1/2 to 1 MW | 0.748 | *** | 0.221 |  | 1.433 | *** | 1.004 | *** |
|  | (0.189) |  | (0.199) |  | (0.316) |  | (0.251) |  |
| 1 to 2 MW | 0.607 | *** | 0.182 |  | 1.026 | *** | 0.924 | *** |
|  | (0.169) |  | (0.178) |  | (0.305) |  | (0.229) |  |
| 2 to 3 MW | 0.305 |  | -0.038 |  | 0.781 | * | 0.271 |  |
|  | (0.173) |  | (0.181) |  | (0.323) |  | (0.257) |  |
| Anomaly Precipitation (mm) | 0.004 | ** | 0.001 |  | -0.001 |  | -0.001 |  |
|  | (0.002) |  | (0.002) |  | (0.002) |  | (0.002) |  |
| Elevation | -0.001 |  | -0.004 | *** | -0.002 |  | -0.002 | * |
|  | (0.001) |  | (0.001) |  | (0.001) |  | (0.001) |  |
| Soil Moisture | 0.002 | * | 0.002 | ** | 0.001 |  | 0.003 | ** |
|  | (0.001) |  | (0.001) |  | (0.001) |  | (0.001) |  |
| Intercept | -2.248 | ** | -0.813 |  | -3.776 | *** | -3.410 | *** |
|  | (0.712) |  | (0.742) |  | (1.104) |  | (0.950) |  |
| Number of observations | 1411. |  | 1411. |  | 1411. |  | 1411. |  |
| χ² | 84.9644261 |  | 136.7668286 |  | 72.3670561 |  | 75.3980393 |  |

Results align closely with the PCA variable findings. Lower-income households show consistently higher odds of experiencing flooding and downstream losses (home loss/homelessness and damaged goods), and informal employment is associated with greater reported impacts. Age and most religion/education covariates exhibit small or imprecise associations, mirroring their limited roles in the WRIS models. The precipitation-anomaly coefficient carries the expected sign but is modest in magnitude and not precisely estimated in all specifications, which is unsurprising given the rarity of severe outcomes once indicators are binarized. Overall, the socioeconomic gradients and the direction of environmental effects are substantively unchanged relative to the PCA approach. These standalone models therefore corroborate that our core conclusions do not depend on the construction of the composite index.

### S.2.3 Aggregated Impact Score

As part of our robustness checks, we replicated our primary regression models using the unweighted, raw aggregated flood impact score as the outcome variable. Table S.4 presents the results from three model specifications: an OLS model without fixed effects Equation (3) shown in Column 1, a fixed-effects model without precipitation adjustment (Column 2), and a fully adjusted fixed-effects model that includes precipitation in May 2022 (Column 3). These models parallel those presented in Table 2 of the main text but use the non-weighted impact score as a sensitivity test to ensure that our findings are not dependent on the weighting scheme.

Across all three models, the results are substantively consistent with those reported in the main analysis using the weighted impact score. Socioeconomic stratifiers—particularly Household Income, Informal Labor Market participation, and Number of Children Under 15—remain statistically significant predictors of reported flood disruption. For instance, in the fixed-effects model with precipitation adjustment (Column 3), respondents earning 1/2 minimum wage or below report impact scores that are 2.085 points higher than the highest income group (p < 0.001), while those earning between 1/2 to 1 and 1 to 2 minimum wages also report significantly elevated scores of 2.016 and 1.774, respectively. These effects are strikingly similar in both magnitude and significance to those in the weighted models, reinforcing the strength and stability of the observed income gradient.

Importantly, the inclusion of precipitation anomalies (Column 3) has minimal effect on the significance or magnitude of most covariates. The precipitation coefficient itself is statistically insignificant, further supporting our earlier interpretation that measured exposure does not fully account for disparities in reported flood impact. Instead, social stratification and household vulnerability appear to be the dominant explanatory factors—whether the outcome is constructed as a weighted or raw score.

Therefore, this sensitivity analysis strongly confirms the robustness of our findings. Regardless of whether flood impact is weighted by severity or treated as a simple additive measure, the same key stratifiers emerge as significant, reinforcing our core argument that flood disruption is socially patterned and mediated by household-level factors.

Table S4: Regression Results of Non-Weighted Reported Impact Score: OLS and Fixed-Effects Models with Demographic, Socioeconomic and Household Stratifiers as well as Precipitation

|  | Main Model | | Fixed Effect Model  Without Anom. Precip. | | Fixed Effect Model  With Anom. Precip. | |
| --- | --- | --- | --- | --- | --- | --- |
| Age (2022) | 0.011 | | 0.003 | | 0.002 | |
| Race (Baseline: White) | (0.029) | | (0.030) | | (0.030) | |
| Non-White | 0.595 | * | 0.320 |  | 0.326 |  |
| Religion (Baseline: Catholic) | (0.285) |  | (0.290) |  | (0.290) |  |
| Non-Pentecostal Evangelical | 0.354 |  | 0.080 |  | 0.072 |  |
|  | (0.361) |  | (0.366) |  | (0.367) |  |
| Pentecostal Evangelical | 0.596 |  | 0.411 |  | 0.416 |  |
|  | (0.359) |  | (0.370) |  | (0.370) |  |
| Other religion | 0.525 |  | 0.226 |  | 0.231 |  |
|  | (0.508) |  | (0.508) |  | (0.508) |  |
| Atheist/Agnostic | 0.139 |  | 0.044 |  | 0.048 |  |
| Respondent’s Mother’s Education (Baseline: Less than High school) | (0.358) |  | (0.366) |  | (0.366) |  |
| No formal education | -0.094 |  | 0.479 |  | 0.478 |  |
|  | (0.563) |  | (0.603) |  | (0.603) |  |
| Highschool | 0.375 |  | 0.102 |  | 0.101 |  |
|  | (0.300) |  | (0.306) |  | (0.307) |  |
| Some College or More | 0.765 | * | 0.336 |  | 0.334 |  |
| Respondent’s Education (Baseline: High School or Less) | (0.388) |  | (0.394) |  | (0.394) |  |
| Some College or More | 0.055 |  | 0.234 |  | 0.239 |  |
| Employment Status (Baseline: Formal Employment) | (0.297) |  | (0.301) |  | (0.301) |  |
| Informal Labor Market | 1.097 | ** | 0.852 | * | 0.844 | * |
|  | (0.361) |  | (0.366) |  | (0.366) |  |
| Out of Labor Market | 0.253 |  | 0.124 |  | 0.124 |  |
|  | (0.316) |  | (0.323) |  | (0.323) |  |
| Current Number of Children Under 15 0.373 | | * | 0.392 | * | 0.390 | * |
| (0.154) | |  | (0.157) |  | (0.157) |  |
| Household Income (Baseline: 3 Minimum Wage or Above)  1/2 MW or Below 1.581 ** | | | 2.079 | *** | 2.085 | *** |
| (0.551) | | | (0.568) |  | (0.568) |  |
| 1/2 to 1 MW 2.021 *** | | | 2.019 | *** | 2.016 | *** |
| (0.426) | | | (0.436) |  | (0.437) |  |
| 1 to 2 MW 1.731 *** | | | 1.773 | *** | 1.774 | *** |
| (0.381) | | | (0.388) |  | (0.388) |  |
| 2 to 3 MW 0.621 | | | 0.834 | * | 0.838 | * |
| (0.394) | | | (0.400) |  | (0.400) |  |
| Anomaly Precipitation (mm) | | |  |  | -0.002 |  |
|  | | |  |  | (0.005) |  |
| Intercept 1.675 | | | 2.332 | * | 2.928 |  |
| (0.979) | | | (1.005) |  | (1.556) |  |
| Number of observations 1417 | | | 1395 |  | 1395 |  |
| F statistic 4.933925 | | | 4.417269 |  | 4.183446 |  |
| R-squared .0565635 | | | .1500407 |  | .1502053 |  |
| Number of absorbed categories | | | 78 |  | 78 |  |
| *** p*<*.001, ** p*<*.01, * p*<*.05 | | |  |  |  |  |


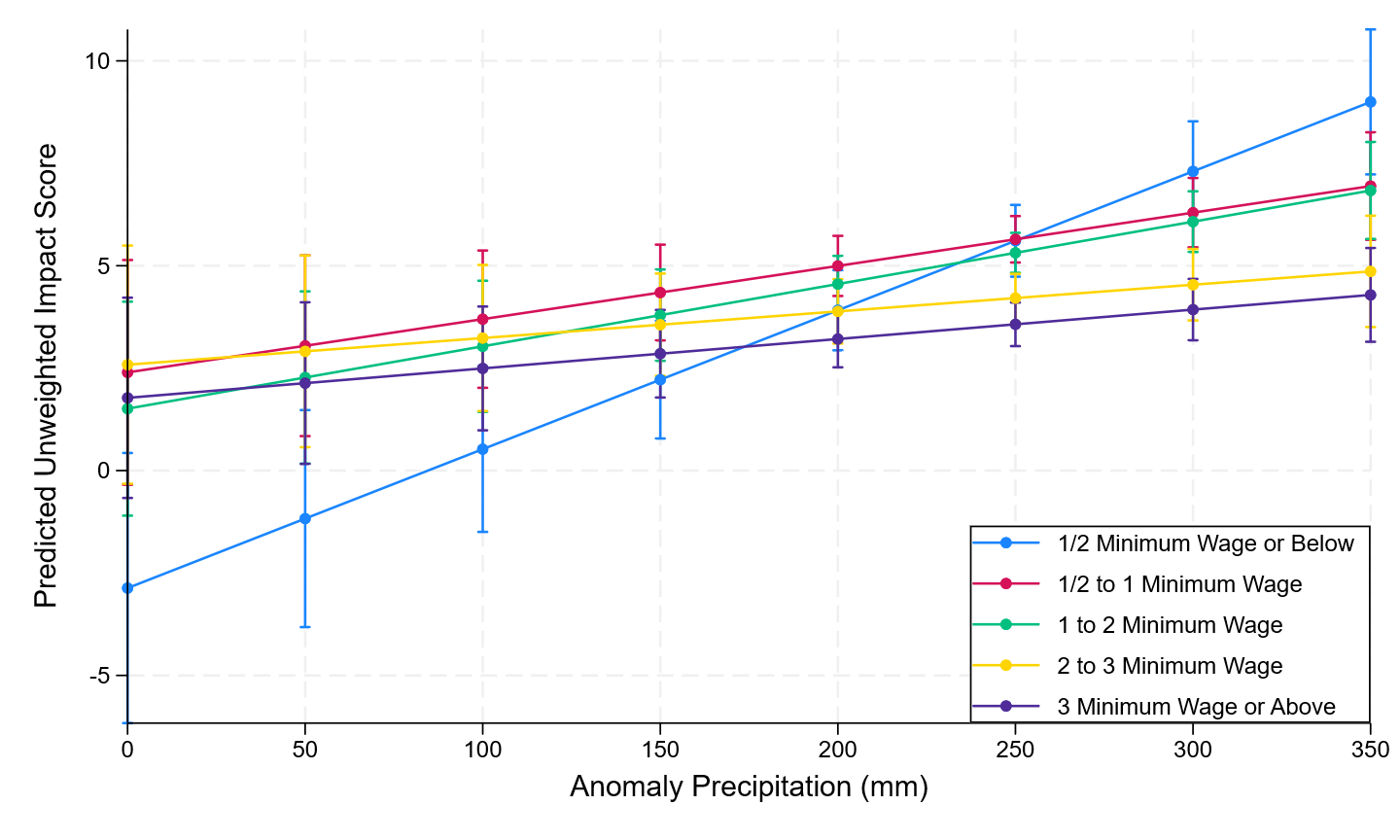


Figure S.1: Sensitivity Analysis: Predicted Aggregate Effect Scores by Anomalous Precipitation and Household Income (OLS Model without Fixed Effects)

*Note:* This figure presents a sensitivity analysis using the Unweighted Effect Score to assess the robustness of the main findings. The predictions are based on the OLS specification of the third model in Table S4. Predicted values are generated from an OLS regression (without municipal fixed effects) including an interaction between household income categories and anomalous precipitation. The Unweighted Effect Score is a composite index of self-reported flood-related disruptions, without applying category weights. Anomalous precipitation refers to deviation from the long-term historical average for May 2022, measured in millimeters (mm). Confidence intervals represent 95\% uncertainty bounds. While some predicted values fall below zero, the actual Unweighted Effect Score is bounded at zero; these negative predictions are a statistical artifact of the linear model. The consistency in trend and stratification patterns between this figure and the main results based on the Weighted Effect Score suggests that the findings are robust to alternate impact score constructions. Income categories correspond to monthly household income in Brazilian minimum wage (MW) ranges, e.g., “1 to 2 Minimum Wage” refers to households earning between one and two times the minimum wage.


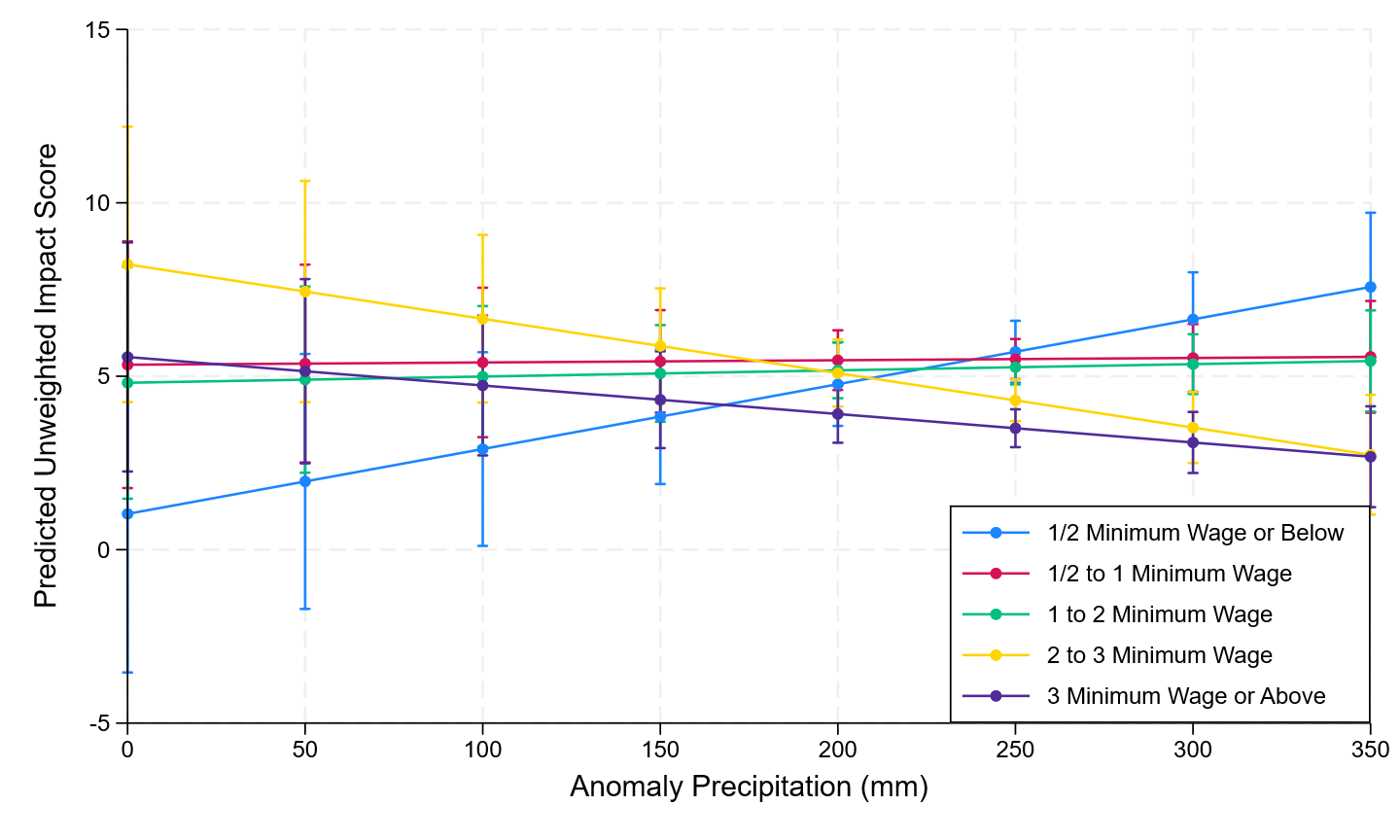


Figure S.2: Sensitivity Analysis: Predicted Aggregate Effect Scores by Anomalous Precipitation and Household Income (Fixed Effects Model)

*Note:* This figure presents a sensitivity analysis using the Unweighted Effect Score to assess the robustness of the main findings under a fixed effects specification. The estimates are generated from the third model in Table S4. Predicted values are generated from a regression model that absorbs municipal-level fixed effects and includes an interaction between household income categories and anomalous precipitation. The Unweighted Effect Score is a composite measure of self-reported flood-related disruptions, constructed without applying weights to individual indicators. Anomalous precipitation refers to deviation from the long-term historical average for May 2022, measured in millimeters (mm). Confidence intervals represent 95\% uncertainty bounds. Although some predicted values fall below zero, the actual Unweighted Effect Score is bounded at zero; negative predictions are a statistical artifact of the linear model. This figure demonstrates that the core stratification patterns and precipitation sensitivity observed with the weighted outcome also emerge with the unweighted version, reinforcing the robustness of the results. Income categories represent household monthly income ranges in Brazilian minimum wage (MW) units.

### S.2.4 Variable Correlations and Multicollinearity Assumption

Variance inflation factors (VIFs) were computed to assess multicollinearity across covariates. All VIF values fall well below the conventional threshold of 5 (or even 2.5 for stricter criteria), indicating no concerning collinearity among the predictors. The mean VIF is 1.39.

Table S5: Variance inflation factors of all variables and OLS models

| **Variable** | **VIF** | **1/VIF** |
| --- | --- | --- |
| Age (2022) | 1.29 | 0.776 |
| Race: Non-White | 1.06 | 0.94 |
| Religion: Non-Pentecostal | 1.25 | 0.801 |
| Religion: Pentecostal | 1.28 | 0.781 |
| Religion: Other | 1.15 | 0.869 |
| Religion: Atheist/Agnostic | 1.26 | 0.791 |
| Mother’s Education: High School | 1.28 | 0.784 |
| Mother’s Education: Some College+ | 1.47 | 0.68 |
| Respondent’s Education: Some College+ | 1.37 | 0.727 |
| Employment: Informal | 1.27 | 0.79 |
| Employment: Out of Labor Force | 1.37 | 0.73 |
| Number of Children < 15 | 1.32 | 0.76 |
| Income: ≤ ½ MW | 1.67 | 0.599 |
| Income: ½–1 MW | 1.93 | 0.519 |
| Income: 1–2 MW | 1.75 | 0.57 |
| Income: 2–3 MW | 1.45 | 0.689 |
| **Mean VIF** | **1.39** | |

### S.2.5 Soil Moisture OLS and Fixed Effects Regressions

Table S6: Alternative measures of hazard-exposure both OLS and Fixed Effects

|  | Soil Moisture (OLS) | | Soil Moisture (FE) | | 6-Day Anomalies (OLS) | | 6-Day Anomalies (FE) | |
| --- | --- | --- | --- | --- | --- | --- | --- | --- |
| Age (2022) | 1.240 | ** | 0.347 |  | -0.017 |  | 0.003 |  |
|  | (0.442) |  | (0.244) |  | (0.009) |  | (0.004) |  |
| Race (Baseline: White) |  |  |  |  |  |  |  |  |
| Non-White | 2.961 |  | 0.528 |  | -0.035 |  | 0.009 |  |
|  | (4.362) |  | (2.389) |  | (0.092) |  | (0.034) |  |
| Religion (Baseline: Catholic) |  |  |  |  |  |  |  |  |
| Non-Pentecostal Evangelical | 7.862 |  | -3.328 |  | -0.205 |  | 0.028 |  |
|  | (5.517) |  | (3.015) |  | (0.117) |  | (0.044) |  |
| Pentecostal Evangelical | 4.374 |  | -1.511 |  | -0.121 |  | 0.018 |  |
|  | (5.528) |  | (3.056) |  | (0.117) |  | (0.044) |  |
| Other religion | -4.555 |  | 2.486 |  | -0.240 |  | -0.047 |  |
|  | (7.848) |  | (4.215) |  | (0.166) |  | (0.061) |  |
| Atheist/Agnostic | 6.573 |  | 1.421 |  | -0.241 | * | -0.001 |  |
|  | (5.487) |  | (3.018) |  | (0.116) |  | (0.044) |  |
| Respondent’s Mother’s Education (Baseline: Less than High school) |  |  |  |  |  |  |  |  |
| Highschool | -2.077 |  | -1.291 |  | -0.259 | ** | 0.002 |  |
|  | (4.518) |  | (2.479) |  | (0.096) |  | (0.036) |  |
| Some College or More | 0.453 |  | 1.801 |  | -0.311 | * | -0.042 |  |
|  | (5.904) |  | (3.221) |  | (0.125) |  | (0.047) |  |
| Respondent’s Education (Baseline: High School or Less) |  |  |  |  |  |  |  |  |
| Some College or More | 2.853 |  | 0.633 |  | 0.157 |  | 0.031 |  |
|  | (4.543) |  | (2.483) |  | (0.096) |  | (0.036) |  |
| Employment Status (Baseline: Formal Employment) |  |  |  |  |  |  |  |  |
| Informal Labor Market | -1.139 |  | 1.432 |  | -0.031 |  | -0.034 |  |
|  | (5.527) |  | (3.018) |  | (0.117) |  | (0.044) |  |
| Out of Labor Market | -0.607 |  | 0.079 |  | 0.156 |  | 0.005 |  |
|  | (4.843) |  | (2.669) |  | (0.102) |  | (0.039) |  |
| Current Number of Children Under 15 | -4.223 |  | -0.611 |  | 0.025 |  | -0.007 |  |
|  | (2.358) |  | (1.296) |  | (0.050) |  | (0.019) |  |
| Household Income (Baseline: 3 Minimum Wage or Above) |  |  |  |  |  |  |  |  |
| 1/2 MW or Below | -0.944 |  | -1.843 |  | -0.039 |  | 0.028 |  |
|  | (8.425) |  | (4.670) |  | (0.178) |  | (0.067) |  |
| 1/2 to 1 MW | 13.632 | * | 6.756 |  | -0.056 |  | 0.039 |  |
|  | (6.526) |  | (3.599) |  | (0.138) |  | (0.052) |  |
| 1 to 2 MW | 9.252 |  | 2.491 |  | -0.023 |  | 0.079 |  |
|  | (5.835) |  | (3.200) |  | (0.123) |  | (0.046) |  |
| 2 to 3 MW | -1.451 |  | -0.071 |  | -0.026 |  | 0.154 | ** |
|  | (6.018) |  | (3.291) |  | (0.127) |  | (0.048) |  |
| Elevation | -0.278 | *** | -0.293 | *** | -0.001 | ** | 0.006 | *** |
|  | (0.016) |  | (0.034) |  | (0.000) |  | (0.000) |  |
| Intercept | 425.306 | *** | 458.022 | *** | 6.084 | *** | 4.828 | *** |
|  | (15.046) |  | (8.525) |  | (0.318) |  | (0.123) |  |
| Number of observations | 1411. |  | 1389. |  | 1411. |  | 1389. |  |
| F statistic | 20.9712107 |  | 5.0812613 |  | 1.6491056 |  | 10.1532893 |  |
| R-squared | 0.20377 |  | 0.7869564 |  | 0.0197284 |  | 0.8794434 |  |
| DF for absorbed effect | 73 |  | 77. |  |  |  | 77. |  |
| *** p<.001, ** p<.01, * p<.05 |  |  |  |  |  |  |  |  |

To verify that our results are not sensitive to how rainfall exposure is defined, we re-estimated the main specification replacing the monthly precipitation anomaly with two alternatives that target near-surface hydrologic conditions and short, intense events: (i) a satellite-based soil-moisture index and (ii) a 6-day precipitation anomaly centered on the flooding period. Each exposure was standardized, and we retained the full covariate set used in the main models (demographics, education, employment, household income, number of children, and environmental controls) with heteroskedasticity-robust standard errors; municipality fixed effects were included in FE columns.

Across both alternatives, the pattern of coefficients mirrors the monthly-anomaly results. Socioeconomic gradients remain: lower-income categories and informal labor are associated with higher reported impacts, while education differences are small. The exposure variables point in the expected direction—soil moisture is positively associated with reported impacts in OLS and attenuates with fixed effects; the 6-day anomaly yields smaller, less precise effects—but the qualitative conclusions are unchanged. Elevation enters negatively, as in the main results, and model fit is comparable. Taken together, these exercises indicate that our conclusions do not hinge on using a monthly rainfall anomaly: results are robust to hydrologically proximate (soil moisture) and short-window (6-day) measures of exposure.

## S.3 Robustness Check: Mediation with Treatment–Mediator Interaction

To test whether our mediation findings depend on the no-interaction assumption, we re-estimated the model using Stata’s mediate command while including an interaction between the treatment (household income, 5 categories; reference = ≥3 MW) and the mediator (water-supply outage, binary). The outcome is the WRIS index. We report natural indirect effects (NIE), natural direct effects (NDE), and total effects (TE) with robust standard errors (Table S7).

Translating to proportions mediated (NIE/TE), water outages account for roughly 16.3% (≤½ MW), 14.6% (½–1 MW), 14.0% (1–2 MW), and 3.9% (2–3 MW; not statistically different from zero). Thus, even when the treatment–mediator interaction is explicitly modeled, the indirect pathway remains meaningfully different from zero for the two lowest income groups and sizeable for the 1–2 MW group, reinforcing the role of piped-water unreliability in amplifying flood impacts among lower-income households.

These results reaffirm our earlier conclusion that water infrastructure disruptions mediate a meaningful portion of the relationship between household income and reported flood impact. Importantly, the significance of the mediation pathway persists even when the treatment–mediator interaction is explicitly modeled. This robustness check strengthens confidence in the stability of our findings, particularly with respect to the role of piped water reliability in amplifying the impact of flooding for lower-income households.

*Table S7: Mediation Analysis with Treatment–Mediator Interaction*

| **Income Group (vs Ref ≥ 3+ MW)** | **NIE** | **NDE** | **TE** | **Proportion Mediated** |
| --- | --- | --- | --- | --- |
| ≤ 1/2 MW (1 vs 5) | 0.111 | 0.569** | 0.680** | 16.30% |
|  | -0.064 | -0.213 | -0.197 |  |
| 1/2–1 MW (2 vs 5) | 0.103* | 0.601*** | 0.704*** | 14.60% |
|  | -0.044 | -0.134 | -0.13 |  |
| 1–2 MW (3 vs 5) | 0.077* | 0.471*** | 0.547*** | 14.00% |
|  | -0.032 | -0.109 | -0.109 |  |
| 2–3 MW (4 vs 5) | 0.007 | 0.167 | 0.174 | 3.90% |
|  | -0.016 | -0.103 | -0.1 |  |

**Notes:** Entries are coefficients with robust standard errors in parentheses. Stars denote significance (* p<0.05, ** p<0.01, *** p<0.001). Proportion Mediated = NIE / TE × 100.

## S.4.1 Replication of Mediation Findings via Structural Equation Modeling

To assess the robustness of our initial mediation findings—originally estimated using the mediate command—we re-estimated the model using structural equation modeling xi:sem, deriving indirect effects through the product-of-coefficients approach via nlcom. In this specification, Household Income is modeled as a five-category treatment variable, Water Supply Outages as the binary mediator, and the Weighted Reported Impact Score as the continuous outcome.

All four lower-income groups (below the highest category of 3 or more minimum wages) exhibit statistically significant indirect effects, ranging from 0.024 to 0.053 points ($p < 0.05$). The strongest mediated effects are observed among the lowest two income tiers—those earning up to 1 minimum wage—highlighting the degree to which infrastructural vulnerability contributes to heightened flood impact in more disadvantaged groups.These findings reinforce the interpretation that infrastructural marginalization—captured here through the binary indicator of water supply disruptions—represents a meaningful mechanism through which income-based disparities in flood impacts are generated. The significance of these effects holds even under full-information maximum likelihood estimation and relaxed assumptions about error correlation.

*Table S8: SEM based mediation approach*

| **Income Group** | **Indirect effect (NIE)** | **Std. Err.** | **z** | **p-value** | **[95% CI]** |
| --- | --- | --- | --- | --- | --- |
| ≤ 1/2 MW (1 vs 5 ref) | 0.085*** | 0.026 | 3.26 | 0.001 | 0.034, 0.136 |
| 1/2–1 MW (2 vs 5 ref) | 0.085*** | 0.024 | 3.60 | <0.001 | 0.039, 0.132 |
| 1–2 MW (3 vs 5 ref) | 0.059** | 0.019 | 3.17 | 0.002 | 0.022, 0.095 |
| 2–3 MW (4 vs 5 ref) | 0.039* | 0.017 | 2.34 | 0.019 | 0.006, 0.072 |

## S.4.2 Alternative SEM Specification: Number of Children as Predictor

To assess whether household infrastructure mediates the relationship between household composition and reported flood impact, we conducted a causal mediation analysis using the number of children under 15 as a continuous treatment variable. Specifically, we tested whether the effect of having additional children on the reported impact score operates in part through disruptions in water supply. We employed the same specification described in Section 5, using Stata’s mediat} command with the binary indicator of water outages as the mediator.

These findings suggest that while household composition—particularly the number of dependents—is a key predictor of perceived flood disruption, its effect does not appear to operate through household-level water outages. Rather, this relationship likely reflects other mechanisms of vulnerability, such as caregiving burden, resource strain, or physical mobility constraints during disasters. In contrast to income, where infrastructure explained a meaningful share of the disparities in flood impact, household composition appears to exert its influence through different pathways not captured by our water outage proxy.

Table S9: Causal Mediation Analysis of Children Under 15 and Reported Flood Impact via Water Outages

| ***Children (vs 0)*** | ***NIE*** | ***NDE*** | ***TE*** | ***% Mediated*** |
| --- | --- | --- | --- | --- |
| *1 vs 0* | *0.008 (0.007)* | *0.167** (0.057)* | *0.175** (0.058)* | *4.5%* |
| *2 vs 0* | *0.015 (0.014)* | *0.334** (0.113)* | *0.350** (0.115)* | *4.3%* |
| *3 vs 0* | *0.022 (0.024)* | *0.502** (0.170)* | *0.523** (0.173)* | *4.1%* |
| *4 vs 0* | *0.027 (0.037)* | *0.669** (0.227)* | *0.696** (0.232)* | *3.9%* |
| *5 vs 0* | *0.032 (0.054)* | *0.836** (0.284)* | *0.868** (0.291)* | *3.7%* |
| *6 vs 0* | *0.035 (0.075)* | *1.003** (0.340)* | *1.039** (0.351)* | *3.4%* |

*Note*: NIE = Natural Indirect Effect; NDE = Natural Direct Effect. Proportion Mediated = NIE / TE, shown as a percentage. Standard errors are shown in parentheses beneath coefficients. Estimates are based on Stata’s mediate command. Significance is based on two-tailed tests. *Significance levels*: ^*^ *p <* 0*.*05, ^**^ *p <* 0*.*01, ^***^ *p <* 0*.*001.

## S.5 Standalone Indicator Mediation Analysis

Similar to the PCA mediation analysis, we note that all NIEs are significant across the four indicators reported by respondents.

Table S10: Causal Mediation Analysis of dichotomous flooding reports

| **Income Group** | **NIE** | **NDE** | **TE** | **% Mediated** |
| --- | --- | --- | --- | --- |
| ≤ 1/2 MW (1 vs 5 ref) | 0.021* (0.008) | −0.117* (0.057) | −0.096† (0.057) | 21.7% |
| 1/2–1 MW (2 vs 5 ref) | 0.018** (0.006) | 0.009 (0.044) | 0.027 (0.043) | 65.5% |
| 1–2 MW (3 vs 5 ref) | 0.012* (0.005) | 0.015 (0.040) | 0.027 (0.039) | 44.8% |
| 2–3 MW (4 vs 5 ref) | 0.010* (0.004) | −0.037 (0.041) | −0.027 (0.041) | 36.4% |

*Notes:* Outcome = flooding at/near home (logit). Mediator = water supply outage (logit). Treatment = household income (ref = ≥ 3 MW). Robust SEs in parentheses. Stars: * p<0.05, ** p<0.01, *** p<0.001, † p<0.10. Proportion mediated = NIE/TE × 100 (Absolute Value).

Table S11: Causal Mediation Analysis of dichotomous Lack of Connection reports

| **Income Group** | **NIE** | **NDE** | **TE** | **% Mediated** |
| --- | --- | --- | --- | --- |
| ≤ 1/2 MW (1 vs 5 ref) | 0.029** (0.010) | 0.110† (0.057) | 0.138* (0.057) | 20.8% |
| 1/2–1 MW (2 vs 5 ref) | 0.027*** (0.008) | 0.154*** (0.045) | 0.181*** (0.044) | 14.8% |
| 1–2 MW (3 vs 5 ref) | 0.019** (0.006) | 0.126*** (0.040) | 0.145*** (0.040) | 12.8% |
| 2–3 MW (4 vs 5 ref) | 0.015* (0.006) | 0.044 (0.041) | 0.059 (0.041) | 25.1% |

*Notes:* Outcome = lack of water/electricity/internet/cell connection (logit). Mediator = water supply outage (logit). Treatment = household income (ref = ≥ 3 MW). Robust SEs in parentheses. Stars: * p<0.05, ** p<0.01, *** p<0.001, † p<0.10. Proportion mediated = NIE/TE × 100 (Absolute Value).

Table S12: Causal Mediation Analysis of dichotomous Homelessness reports

| Income Group | NIE | NDE | TE | % Mediated |
| --- | --- | --- | --- | --- |
| ≤ 1/2 MW (1 vs 5 ref) | 0.019* (0.008) | 0.112** (0.037) | 0.131** (0.039) | 14.8% |
| 1/2–1 MW (2 vs 5 ref) | 0.019** (0.007) | 0.122*** (0.027) | 0.141*** (0.028) | 13.8% |
| 1–2 MW (3 vs 5 ref) | 0.010* (0.004) | 0.072** (0.021) | 0.082*** (0.022) | 12.5% |
| 2–3 MW (4 vs 5 ref) | 0.006* (0.003) | 0.043† (0.022) | 0.050* (0.023) | 12.9% |

*Notes:* Outcome = loss of home / homelessness (logit). Mediator = water supply outage (logit). Treatment = household income (ref = ≥ 3 MW). Robust SEs in parentheses. Stars: * p<0.05, ** p<0.01, *** p<0.001, † p<0.10. Proportion mediated = NIE/TE × 100 (Absolute Value).

Table S13: Causal Mediation Analysis of dichotomous Loss of goods reports

| **Income Group** | **NIE** | **NDE** | **TE** | **% Mediated** |
| --- | --- | --- | --- | --- |
| ≤ 1/2 MW (1 vs 5 ref) | 0.021* (0.009) | 0.163*** (0.046) | 0.183*** (0.048) | 11.2% |
| 1/2–1 MW (2 vs 5 ref) | 0.018** (0.007) | 0.129*** (0.033) | 0.147*** (0.034) | 12.5% |
| 1–2 MW (3 vs 5 ref) | 0.012* (0.005) | 0.113*** (0.028) | 0.125*** (0.029) | 9.5% |
| 2–3 MW (4 vs 5 ref) | 0.006* (0.003) | 0.014 (0.026) | 0.020 (0.026) | 29.7% |

*Notes:* Outcome = loss/damage of goods (logit). Mediator = water supply outage (logit). Treatment = household income (ref = ≥ 3 MW). Robust SEs in parentheses. Stars: * p<0.05, ** p<0.01, *** p<0.001, † p<0.10. Proportion mediated = NIE/TE × 100 (Absolute Value).

## S.6 Attrition Analysis

|  | Attrition (Logit) |
| --- | --- |
| Age in Wave 2 | -0.017 |
|  | (0.009) |
| Household Income in Wave 2 |  |
| 1/2 to 1 Minimum Wage | -0.113 |
|  | (0.135) |
| 1 to 2 Minimum Wage | 0.026 |
|  | (0.141) |
| 2 to 3 Minimum Wage | -0.115 |
|  | (0.194) |
| 3 Minimum Wage or Above | -0.256 |
|  | (0.132) |
| Education (Ref: Highschool or Less) |  |
| Incomplete college or more | -0.022 |
|  | (0.091) |
| Intercept | 0.110 |
|  | (0.274) |
| Number of observations | 2507. |
| χ² | 11.5283363 |
| *** p<.001, ** p<.01, * p<.05 |  |

## S.7 Elevation adjusted OLS Exposure

Table S15: OLS Exposure with elevation

|  | OLS Exposure | |
| --- | --- | --- |
| Age (2022) | -0.435 | * |
|  | (0.222) |  |
| Race (Baseline: White) |  |  |
| Non-White | 3.343 |  |
|  | (2.186) |  |
| Religion (Baseline: Catholic) |  |  |
| Non-Pentecostal Evangelical | -3.685 |  |
|  | (2.764) |  |
| Pentecostal Evangelical | 0.255 |  |
|  | (2.769) |  |
| Other religion | 6.480 |  |
|  | (3.932) |  |
| Atheist/Agnostic | 1.149 |  |
|  | (2.749) |  |
| Respondent’s Mother’s Education (Baseline: Less than High school) |  |  |
| Highschool | 1.118 |  |
|  | (2.263) |  |
| Some College or More | -2.230 |  |
|  | (2.958) |  |
| Respondent’s Education (Baseline: High School or Less) |  |  |
| Some College or More | 3.153 |  |
|  | (2.276) |  |
| Employment Status (Baseline: Formal Employment) |  |  |
| Informal Labor Market | 0.715 |  |
|  | (2.769) |  |
| Out of Labor Market | 0.564 |  |
|  | (2.427) |  |
| Current Number of Children Under 15 | -0.654 |  |
|  | (1.181) |  |
| Household Income (Baseline: 3 Minimum Wage or Above) |  |  |
| 1/2 MW or Below | -0.684 |  |
|  | (4.221) |  |
| 1/2 to 1 MW | -0.121 |  |
|  | (3.270) |  |
| 1 to 2 MW | 2.582 |  |
|  | (2.923) |  |
| 2 to 3 MW | 1.193 |  |
|  | (3.015) |  |
| elevation | -0.268 | *** |
|  | (0.008) |  |
| Intercept | 268.496 | *** |
|  | (7.538) |  |
| Number of observations | 1411. |  |
| R-squared | 0.479643 |  |
| F statistic | ######## |  |
| *** p<.001, ** p<.01, * p<.05 |  |  |

## S.8 Elevation adjusted OLS Exposure

Table S16: Leave-one-municipality-out estimation

|  | OLS Exposure | | OLS Impact | | FE Exposure | | FE Impact | | FE Impact (Adjusted) | |
| --- | --- | --- | --- | --- | --- | --- | --- | --- | --- | --- |
| Age (2022) | -0.746 | * | 0.005 |  | -0.246 |  | 0.004 |  | 0.004 |  |
|  | (0.296) |  | (0.011) |  | (0.234) |  | (0.012) |  | (0.012) |  |
| Race (Baseline: White) |  |  |  |  |  |  |  |  |  |  |
| Non-White | 4.364 |  | 0.224 | * | 2.589 |  | 0.156 |  | 0.158 |  |
|  | (2.911) |  | (0.108) |  | (2.277) |  | (0.113) |  | (0.113) |  |
| Religion (Baseline: Catholic) |  |  |  |  |  |  |  |  |  |  |
| Non-Pentecostal Evangelical | -3.012 |  | 0.051 |  | -3.960 |  | -0.053 |  | -0.063 |  |
|  | (3.697) |  | (0.137) |  | (2.886) |  | (0.143) |  | (0.143) |  |
| Pentecostal Evangelical | 0.722 |  | 0.124 |  | 2.750 |  | 0.044 |  | 0.049 |  |
|  | (3.643) |  | (0.135) |  | (2.879) |  | (0.143) |  | (0.143) |  |
| Other religion | 11.086 | * | 0.039 |  | 4.096 |  | -0.112 |  | -0.112 |  |
|  | (5.530) |  | (0.205) |  | (4.214) |  | (0.209) |  | (0.209) |  |
| Atheist/Agnostic | 2.944 |  | 0.008 |  | 3.177 |  | -0.035 |  | -0.030 |  |
|  | (3.661) |  | (0.136) |  | (2.877) |  | (0.142) |  | (0.143) |  |
| Respondent’s Mother’s Education (Baseline: Less than High school) |  |  |  |  |  |  |  |  |  |  |
| Highschool | 0.624 |  | 0.049 |  | -1.718 |  | -0.129 |  | -0.127 |  |
|  | (3.041) |  | (0.112) |  | (2.379) |  | (0.118) |  | (0.118) |  |
| Some College or More | -0.387 |  | 0.285 |  | -0.477 |  | 0.051 |  | 0.051 |  |
|  | (4.058) |  | (0.151) |  | (3.164) |  | (0.157) |  | (0.157) |  |
| Respondent’s Education (Baseline: High School or Less) |  |  |  |  |  |  |  |  |  |  |
| Some College or More | 3.890 |  | 0.118 |  | 2.281 |  | 0.222 |  | 0.225 |  |
|  | (3.113) |  | (0.116) |  | (2.434) |  | (0.121) |  | (0.121) |  |
| Employment Status (Baseline: Formal Employment) |  |  |  |  |  |  |  |  |  |  |
| Informal Labor Market | 1.773 |  | 0.502 | *** | -4.105 |  | 0.419 | ** | 0.417 | ** |
|  | (3.717) |  | (0.138) |  | (2.901) |  | (0.144) |  | (0.144) |  |
| Out of Labor Market | 1.518 |  | 0.132 |  | 0.426 |  | 0.119 |  | 0.122 |  |
|  | (3.250) |  | (0.121) |  | (2.564) |  | (0.127) |  | (0.127) |  |
| Current Number of Children Under 15 | -0.804 |  | 0.136 | * | -1.452 |  | 0.149 | * | 0.143 | * |
|  | (1.612) |  | (0.060) |  | (1.267) |  | (0.063) |  | (0.063) |  |
| Household Income (Baseline: 3 Minimum Wage or Above) |  |  |  |  |  |  |  |  |  |  |
| 1/2 MW or Below | -5.445 |  | 0.816 | *** | -0.101 |  | 1.052 | *** | 1.051 | *** |
|  | (5.721) |  | (0.212) |  | (4.570) |  | (0.226) |  | (0.226) |  |
| 1/2 to 1 MW | -3.434 |  | 0.712 | *** | -4.544 |  | 0.700 | *** | 0.700 | *** |
|  | (4.438) |  | (0.165) |  | (3.505) |  | (0.174) |  | (0.174) |  |
| 1 to 2 MW | 0.870 |  | 0.479 | ** | -1.949 |  | 0.498 | ** | 0.496 | ** |
|  | (4.018) |  | (0.150) |  | (3.161) |  | (0.156) |  | (0.157) |  |
| 2 to 3 MW | 0.319 |  | 0.240 |  | 1.190 |  | 0.329 | * | 0.326 | * |
|  | (4.126) |  | (0.153) |  | (3.245) |  | (0.161) |  | (0.161) |  |
| elevation | -0.270 | *** |  |  | -0.037 |  | -0.002 |  | -0.002 |  |
|  | (0.009) |  |  |  | (0.029) |  | (0.001) |  | (0.002) |  |
| Anomaly Precipitation (mm) |  |  |  |  |  |  |  |  | -0.002 |  |
|  |  |  |  |  |  |  |  |  | (0.002) |  |
| Soil Moisture |  |  |  |  |  |  |  |  | -0.001 |  |
|  |  |  |  |  |  |  |  |  | (0.001) |  |
| Intercept | 277.388 | *** | -1.139 | ** | 253.164 | *** | -0.794 |  | 0.222 |  |
|  | (10.131) |  | (0.375) |  | (8.296) |  | (0.411) |  | (0.940) |  |
| Number of observations | 1010. |  | 1010. |  | 957. |  | 957. |  | 957. |  |
| R-squared | 0.5021069 |  | 0.0707161 |  | 0.7132497 |  | 0.1607261 |  | 0.1621598 |  |
| F statistic | 58.8468023 |  | 4.7227956 |  | 1.3808874 |  | 4.5584121 |  | 4.1569568 |  |
| df_a |  |  |  |  | 44. |  | 44. |  | 44. |  |
| *** p<.001, ** p<.01, * p<.05 |  |  |  |  |  |  |  |  |  |  |

Table S17: Recife Only Estimations

|  | OLS Exposure | | OLS Impact | | FE Exposure | | FE Impact | | FE Impact (Adjusted) | |
| --- | --- | --- | --- | --- | --- | --- | --- | --- | --- | --- |
| Age (2022) | -0.246 |  | 0.008 |  | -0.246 |  | 0.008 |  | 0.008 |  |
|  | (0.299) |  | (0.014) |  | (0.299) |  | (0.014) |  | (0.014) |  |
| Race (Baseline: White) |  |  |  |  |  |  |  |  |  |  |
| Non-White | 3.770 |  | 0.211 |  | 3.770 |  | 0.211 |  | 0.217 |  |
|  | (3.089) |  | (0.144) |  | (3.089) |  | (0.144) |  | (0.144) |  |
| Religion (Baseline: Catholic) |  |  |  |  |  |  |  |  |  |  |
| Non-Pentecostal Evangelical | -5.784 |  | -0.103 |  | -5.784 |  | -0.103 |  | -0.112 |  |
|  | (3.816) |  | (0.178) |  | (3.816) |  | (0.178) |  | (0.178) |  |
| Pentecostal Evangelical | 2.776 |  | 0.083 |  | 2.776 |  | 0.083 |  | 0.084 |  |
|  | (3.864) |  | (0.180) |  | (3.864) |  | (0.180) |  | (0.180) |  |
| Other religion | 2.814 |  | -0.203 |  | 2.814 |  | -0.202 |  | -0.202 |  |
|  | (5.253) |  | (0.245) |  | (5.253) |  | (0.245) |  | (0.245) |  |
| Atheist/Agnostic | 4.656 |  | -0.134 |  | 4.656 |  | -0.134 |  | -0.119 |  |
|  | (3.848) |  | (0.179) |  | (3.848) |  | (0.180) |  | (0.180) |  |
| Respondent’s Mother’s Education (Baseline: Less than High school) |  |  |  |  |  |  |  |  |  |  |
| Highschool | -2.595 |  | -0.169 |  | -2.595 |  | -0.169 |  | -0.167 |  |
|  | (3.147) |  | (0.147) |  | (3.147) |  | (0.147) |  | (0.147) |  |
| Some College or More | 1.374 |  | 0.190 |  | 1.374 |  | 0.190 |  | 0.192 |  |
|  | (4.268) |  | (0.199) |  | (4.268) |  | (0.199) |  | (0.199) |  |
| Respondent’s Education (Baseline: High School or Less) |  |  |  |  |  |  |  |  |  |  |
| Some College or More | 2.682 |  | 0.304 | * | 2.682 |  | 0.304 | * | 0.314 | * |
|  | (3.195) |  | (0.149) |  | (3.195) |  | (0.149) |  | (0.149) |  |
| Employment Status (Baseline: Formal Employment) |  |  |  |  |  |  |  |  |  |  |
| Informal Labor Market | -4.025 |  | 0.529 | ** | -4.025 |  | 0.529 | ** | 0.534 | ** |
|  | (3.789) |  | (0.177) |  | (3.789) |  | (0.177) |  | (0.177) |  |
| Out of Labor Market | -0.674 |  | 0.238 |  | -0.674 |  | 0.238 |  | 0.238 |  |
|  | (3.387) |  | (0.158) |  | (3.387) |  | (0.158) |  | (0.158) |  |
| Current Number of Children Under 15 | -2.017 |  | 0.201 | ** | -2.017 |  | 0.201 | ** | 0.194 | * |
|  | (1.657) |  | (0.077) |  | (1.657) |  | (0.077) |  | (0.077) |  |
| Household Income (Baseline: 3 Minimum Wage or Above) |  |  |  |  |  |  |  |  |  |  |
| 1/2 MW or Below | 2.952 |  | 1.166 | *** | 2.952 |  | 1.166 | *** | 1.151 | *** |
|  | (6.249) |  | (0.291) |  | (6.249) |  | (0.292) |  | (0.292) |  |
| 1/2 to 1 MW | -5.014 |  | 0.698 | ** | -5.014 |  | 0.698 | ** | 0.688 | ** |
|  | (4.650) |  | (0.217) |  | (4.650) |  | (0.217) |  | (0.217) |  |
| 1 to 2 MW | -1.310 |  | 0.596 | ** | -1.310 |  | 0.596 | ** | 0.585 | ** |
|  | (4.209) |  | (0.196) |  | (4.209) |  | (0.196) |  | (0.196) |  |
| 2 to 3 MW | 3.196 |  | 0.374 |  | 3.196 |  | 0.374 |  | 0.358 |  |
|  | (4.401) |  | (0.205) |  | (4.401) |  | (0.205) |  | (0.206) |  |
| elevation | 0.014 |  |  |  | 0.014 |  | 0.000 |  | -0.001 |  |
|  | (0.046) |  |  |  | (0.046) |  | (0.002) |  | (0.002) |  |
| Anomaly Precipitation (mm) |  |  |  |  |  |  |  |  | -0.002 |  |
|  |  |  |  |  |  |  |  |  | (0.002) |  |
| Soil Moisture |  |  |  |  |  |  |  |  | -0.003 |  |
|  |  |  |  |  |  |  |  |  | (0.002) |  |
| Intercept | 262.277 | *** | -1.022 | * | 262.277 | *** | -1.021 | * | 1.186 |  |
|  | (10.551) |  | (0.490) |  | (10.551) |  | (0.492) |  | (1.415) |  |
| Number of observations | 668. |  | 668. |  | 668. |  | 668. |  | 668. |  |
| F statistic | 1.2593719 |  | 4.2824041 |  | 1.2593719 |  | 4.0243508 |  | 3.7650465 |  |
| R-squared | 0.0318871 |  | 0.0952282 |  | 0.0318871 |  | 0.0952292 |  | 0.0994195 |  |
| *** p<.001, ** p<.01, * p<.05 |  |  |  |  |  |  |  |  |  |  |
